# A structure-based *in silico* analysis of the Kell blood group system

**DOI:** 10.3389/fimmu.2024.1452637

**Published:** 2024-12-06

**Authors:** Gabriele Mayr, Maike Bublitz, Tim A. Steiert, Britt-Sabina Löscher, Michael Wittig, Hesham ElAbd, Christoph Gassner, Andre Franke

**Affiliations:** ^1^ Institute of Clinical Molecular Biology, University Hospital Schleswig-Holstein (UKSH) and Christian-Albrechts-University of Kiel, Kiel, Germany; ^2^ Institute of Translational Medicine, Faculty of Medical Sciences, Private University in the Principality of Liechtenstein (UFL), Triesen, Liechtenstein

**Keywords:** Kell blood group system, protein structural modeling, KEL missense variants, antigens, protein variants analysis, phenotype, epitope prediction, antigenicity prediction

## Abstract

Kell is one of the most complex blood group systems, with a highly polymorphic genetic background. Extensive allelic variations in the *KEL* gene affect the encoded erythrocyte surface protein Kell. Genetic variants causing aberrant splicing, premature termination of protein translation, or specific amino acid exchanges lead to a variety of different phenotypes with altered Kell expression levels or changes in the antigenic properties of the Kell protein. Using an *in silico* structural model of the Kell protein, we analyzed the biophysical and structural context of all full-length Kell variants of known phenotype. The results provided insights regarding the 3D co-localization of antigenic Kell variants and led us to suggest several conformational epitopes on the Kell protein surface. We found a number of correlations between the properties of individual genetic variants in the Kell protein and their respective serological phenotypes, which we used as a search filter to predict potentially new immunogenic Kell variants from an in-house whole exome sequencing dataset of 19,772 exomes. Our analysis workflow and results aid blood group serologists in predicting whether a newly identified Kell genetic variant may result in a specific phenotype.

## 1 Introduction

The Kell blood group system (ISBT 006) currently has 38 antigens recognized by the ISBT Red Cell Immunogenetics and Blood Group Terminology Working Party (1). In terms of the number of antigens, this makes it the third most complex blood group system after Rh and MNS with 56 and 50 antigens, respectively. The antigens are found on the surface of the Kell protein on the outside of erythrocytes and comprise a broad spectrum of naturally occurring Kell protein variants. Some allelic variants have frequencies in the single-digit percentage range and may therefore be described as polymorphic, while other variants occur much less frequently. Such variations of the Kell blood group protein are of substantial relevance in transfusion medicine due to potential alloimmune reactions following exposure to non-self Kell antigens. Alloantibodies to Kell antigens may cause mild to severe hemolytic transfusion reactions and are causative of hemolytic disease of the fetus and newborn (HDFN), often with anemia and reactions sometimes delayed (2). In a typical Caucasian population, for example, Switzerland with approximately 8.8 Mio inhabitants, 43% of the overall 1,573 antibodies reported by the Swiss hemovigilance system in 2022 belong to the Rh system, followed by anti-Kell antibodies with 15%, antibodies against the MNS system with 13%, and 29% for all other antibody specificities (3). Hence, as inferred from this high incidence of anti-Kell antibodies, Kell may even be regarded as the second most important blood group system in clinical practice after Rh.

The *KEL* gene encodes a single-pass type II membrane glycoprotein with a total length of 732 amino acids (4) and is a member of the M13 family of zinc-binding metallo-endopeptidases. The Kell protein is also known as CD238 and cleaves big endothelin to produce the bioactive vasoconstrictor endothelin-3 (5). Kell blood group antigens localize on the C-terminal extracellular domain that comprises 655 amino acids and can also engage in a covalent linkage to the multi-pass membrane protein XK by a single disulfide bond (6).

### 1.1 Kell blood group antigens

Blood group systems are officially defined as systems of one or more blood group antigens governed by a single gene or complex of two or more closely linked homologous genes. Blood group antigens are defined by antibodies that either occur “naturally” due to incidentally encountering antigens from the environment or are formed as a result of active immunization upon exposure to human red blood cells (RBCs) from another individual (7). The International Society of Blood Transfusion (ISBT) Working Party on Red Cell Immunogenetics and Blood Group Terminology (7) formally records and systematically names all known blood group antigens. The 38 Kell blood group antigens recognized by the ISBT are KEL1 through KEL41 (with KEL8, 9, and 15 being obsolete), of which KEL1 (also known as K), KEL2 (k), KEL3 (Kp^a^), KEL4 (Kp^b^), KEL5 (Ku), KEL6 (Js^a^), and KEL7 (Js^b^) are most common (8). The different Kell antigens are encoded by specific alleles, which are named in a format that starts either with KEL*01 for variants with positivity for the KEL1 antigen or with KEL*02 for variants with positivity for KEL2 (9). KEL*02 is the most common allele in all populations analyzed as yet; hence, it is formally recognized as the reference allele within the Kell blood group system. Thus, by definition, KEL*02 codes for all 25 known wild-type antigens: KEL2, 4, 5, 7, 11–14, 16, 18–20, 22, 26, 27, 29, 30, 32–38, and 40. Some KEL antigens are antithetical, which means that the protein can exist with one of two variants at the same site, and both variants behave antigenic. The primary structures of two antithetical antigens typically differ from each other by a single amino acid at the same site, and homozygous carriers of the corresponding alleles can each develop antibodies against the other antithetic variant. Therefore, for example, homozygous KEL*02/KEL*02 patients can develop anti-KEL1 antibodies against KEL*01/KEL*01 donor blood and vice versa, and may of course also react against KEL*01/KEL*02 heterozygotes. From a molecular point of view, the rare KEL*01 allele (Caucasian allele frequency approximately 4%) and the frequent wild-type allele KEL*02 (Caucasian allele frequency 94%) code for the two antithetical antigens KEL1 and KEL2, which differ from each other only by the single missense mutation c.578C>T causing a Thr193Met mutation of the Kell protein. In addition to the amino acid exchange at position 193, in this case, the sequence motif recognized for *N*-glycosylation of the protein is affected: Asn191-Arg192-Thr193 of the wild-type KEL2 protein is changed to Asn191-Arg192-Met193 in KEL1, causing a lack of Asn191 glycosylation in KEL1 (10, 11). In other KEL antigens, however, the amino acid substitution alone is usually sufficient for their antigenic properties.

### 1.2 The KEL phenotypes K_mod_ and K_0_

In addition to people that express different KEL antigens (KEL phenotypes), there are so-called K_0_ (or KEL*N) individuals that do not express any Kell protein at all on their RBC membranes. This phenotype is rare, e.g., less than one occurrence is expected per one million inhabitants in Austria (12). K_0_ individuals always carry two parental *KEL* alleles with either missense, nonsense, or splice-site mutations (so-called “null” alleles), abolishing any Kell protein expression. Owing to the rarity of this phenotype, K_0_ patients with anti-KEL alloantibodies are extremely difficult to supply with matching blood. At present, 68 null alleles are listed by the ISBT Working Party for Red Cell Immunogenetics and Blood Group Terminology (7, 9) with 18 of them being caused by different missense mutations.

Apart from KEL and K_0_, there is a third, serologically distinct group of phenotypes within the Kell blood group system, the so-called K_mod_, caused by KEL*M alleles. “Mod” stands for “modified” (13) and refers to a very weak expression of Kell proteins on the surface of the erythrocytes, only detectable by using very sensitive serological methods, e.g., adsorption/elution techniques. For the 25 known K_mod_ phenotypes that result from missense mutations, the various underlying point mutations lead to amino acid substitutions that presumably alter the Kell protein in a way that prevents its transport to the cell surface (14). Individuals with a K_mod_ phenotype are usually heterozygous for KEL*M and KEL*N alleles or, in rare (consanguineous) cases, homozygous or compound heterozygous for KEL*M alleles.

### 1.3 Aims and scope

Considering the extensive knowledge on the genetic background of *KEL* polymorphism, we aimed to elucidate to what extent a computer-aided structural analysis of all known (full-length) Kell protein variants could reveal relationships with their observed clinical phenotypes. Any identified characteristics would be expected not to be applicable to control variants known not to trigger an immune response. Structural modeling has been used in prior descriptions of individual new antigens or variants [KEL (15–18), RHD (19, 20), Lutheran (21), and CROMER (22)], but to the best of our knowledge, a comprehensive correlation analysis of *all* known variants has not been performed for Kell or any other blood group system. In view of the increasing use of high-throughput blood group genotyping and resequencing using next-generation sequencing technologies, especially in blood donors, a computer-aided prediction of the immunogenicity of newly observed genetic variants or their likelihood to present as a K_0_/K_mod_ phenotype would be highly advantageous.

In this study, we therefore collated all KEL blood group alleles currently listed in the respective ISBT table (9) that code for full-length variants of the Kell protein and grouped them as being of either antigenic/immunogenic, unexpressed/null, or diminished/mod phenotype. We then generated a 3D structural model of the Kell protein and investigated whether insight could be obtained on the characteristics of antigenic sites, e.g., epitopes, from 3D localization and physicochemical properties of these missense variants, compared to naturally occurring variants with no reported phenotype. Using quantifiable parameters such as evolutionary conservation scores, solvent accessibility, and individual amino acid side chain properties of all variant sites, we assessed whether mutations causing similar phenotypes have a preference for specific regions within the Kell protein, and whether there is a basis for predicting the phenotypes solely based on these parameters.

## 2 Results

### 2.1 Classification of Kell protein variants

#### 2.1.1 Blood group variants

We first grouped all currently known Kell variants that affect expression levels into the two classes NullV and ModV, corresponding to ISBT phenotypes “K_0_” and “K_mod_”, respectively (**Table 1**). Alleles coding for early stop codons or deletions or those causing aberrant splicing were excluded from the analyses. In addition to alleles that cause the K_0_ or K_mod_ phenotype, 35 known antigens on the Kell protein surface are defined by a single amino acid position, and one antigen (KEL35) is the result of two missense mutations. These Kell antigens can be classified into two main groups, depending on whether they are encoded by the wild-type allele KEL*02 (antigenic wild-type, “AgWT”) or whether they deviate from the wild-type allele (antigenic variant, “AgV”) (**Table 2**). The AgWT antigens can trigger a humoral immune response in individuals negative for the wild-type allele KEL*02. The AgV, in contrast, represents a novel antigen created by substitution of one specific amino acid in relation to the wild-type protein. Amino acid positions that carry antithetical antigens include both of each class, AgWT and AgV. In a number of instances, a deviation from wild-type leads to a loss of the antigen rather than an AgV: these are so-called “minus” alleles and are indicated by a minus in the allele name. In total, the ISBT currently lists 31 alleles defined by missense variants that encode 22 different AgWT, 14 AgV, and 17 “minus” alleles, as listed in **Table 2** together with the respective KEL antigen nomenclature. The AgWT/AgV dataset is additionally listed in more detail in **Supplementary Tables S1, S1A, B**, and **S4**.

**Table 1:** Single or double Kell point mutations that ablate or drastically diminish protein expression levels.

No Kell expression (K_0_ phenotype)			Diminished Kell expression (K_mod_ phenotype)		
Mutation(s)	ISBT allele	Frequency	Mutation(s)	ISBT allele	Frequency
Cys77Phe	KEL*02N.28		Arg86Gln**	KEL*02M.13	
Cys82Arg*	KEL*01N.02		Asp102Glu Pro433Leu	KEL*02M.06	
Cys100Ser	KEL*02N.42		Arg192Pro	KEL*02M.15	
Leu133Pro	KEL*02N.35	1.80×10^−06^	Arg192Gln	KEL*02M.19	1.61×10^−05^
Tyr152Cys	KEL*02N.32	8.99×10^−07^	Thr193Arg	KEL*01M.01	
Ile161Phe	KEL*02N.48	8.99×10^−07^	Leu196Val	KEL*02M.17	1.02×10^−04^
Ala313Thr Arg358Thr	KEL*02N.45		Gly263Arg	KEL*02M.10	4.50×10^−06^
Trp316Cys	KEL*02N.66	1.98×10^−05^	Gly263Glu	KEL*02M.16	
Leu377Pro	KEL*02N.62	8.99×10^−07^	Pro326Leu*	KEL*01M.02	
Phe418Ser	KEL*02N.37	5.08×10^−06^	Leu329Pro	KEL*02M.03	4.50×10^−06^
Gly555Glu	KEL*02N.29		Gln362Lys	KEL*02M.14	3.14×10^−05^
Pro560Ala*	KEL*01N.01		Gly555Val	KEL*02M.18	
Pro560Ala	KEL*02N.18		Ser363Asn	KEL*02M.01	3.54×10^−04^
Val570Met	KEL*02N.59	1.78×10^−05^	Ala423Val	KEL*02M.11	8.31×10^−05^
Gly576Arg	KEL*02N.33	4.24×10^−06^	Arg447Trp*	KEL*01M.03	
Leu611Arg	KEL*02N.38	8.99×10^−07^	Asp497Val	KEL*02M.08	
Ser676Asn	KEL*02N.05	3.63×10^−06^	Ile586Ser	KEL*02M.09	
Arg700Gln**	KEL*02N.34		Tyr588Cys	KEL*02M.07	8.99×10^−07^
			Gly641Arg*	KEL*01M.04	
			Ala645Val*	KEL*01M.05	
			Tyr677Cys	KEL*02M.02	6.08×10^−05^
			Gly703Arg*	KEL*01M.06	
			Gly703Arg	KEL*02M.04	2.37×10^−05^
			Pro704His	KEL*02M.12	1.86×10^−05^
**19 (14)**	**18**		**25 (17)**	**24**	

Variants labeled with one asterisk are K_0_ or K_mod_ alleles that can affect Kell expression in combination with the common variant Thr193Met (KEL*01.01). ** indicates such a combined effect with Arg281Trp (KEL*02.03). One K_0_ (KEL*02N.45) and one K_mod_ (KEL*02M.06) allele are each composed of two variants in cis. Frequencies refer to the MAF in the European population as listed in the gnomAD. Numbers in the last row indicate the total number of mutations and alleles; number in brackets counted only those where a single amino acid exchange alone is responsible for the phenotype.

**Table 2:** Known KEL antigens.

ISBT Kell missense variants			Variant class and respective antigens			
Allele name	Mutation(s)	Frequency	“AgWT”	Antigen	“AgV”	Antigen
KEL*01.01	Thr193Met	4.17×10^−02^	**Thr**	KEL:2	**Met**	KEL:1
KEL*01.02	Thr193Ser	8.99×10^−07^			Ser	KEL:1weak
KEL*02.03	Arg281Trp	1.07×10^−02^	**Arg**	KEL:4	**Trp**	KEL:3
KEL*02.21	Arg281Gln	1.02×10^−05^			**Gln**	KEL:21
KEL*02.06	Leu597Pro	3.58×10^−04^	**Leu**	KEL:7	**Pro**	KEL:6
KEL*02.10	Glu494Val	7.12×10^−05^			Val	KEL:10
KEL*02.17	Val302Ala	1.88×10^−03^	**Val**	KEL:11	**Ala**	KEL:17
KEL*02.-12	His548Arg	2.55×10^−04^	His	KEL:12	–	–
KEL*02.-13	Leu329Pro	4.50×10^−06^	Leu	KEL:13	–	–
KEL*02.-14.1	Arg180Cys	1.61×10^−05^	**Arg**	KEL:14	**-**	–
KEL*02.-14.2	Arg180His	1.36×10^−05^				
KEL*02.24	Arg180Pro	2.70×10^−06^			**Pro**	KEL:24
KEL*02.-18.1	Arg130Trp	4.15×10^−05^	Arg	KEL:18	–	–
KEL*02.-18.2	Arg130Gln	2.37×10^−05^			–	–
KEL*02.-19	Arg492Gln	3.44×10^−04^	Arg	KEL:19	–	–
KEL*02.-22	Ala322Val	2.03×10^−05^	Ala	KEL:22	–	–
KEL*02.23	Gln382Arg		–	–	Arg	KEL:23
KEL*02.25	Arg248Gln	1.61×10^−05^	–	–	Gln	KEL:25
KEL*02.-26	Arg406Gln	8.48×10^−06^	Arg	KEL:26	–	–
KEL*02.-27	Glu249Lys	8.99×10^−07^	Glu	KEL:27	–	
KEL*02.28	Arg248Trp	8.47×10^−06^	–	–	Trp	KEL:28
KEL*02.-29	Arg623Lys	8.99×10^−07^	Arg	KEL:29	–	–
KEL*02.-30	Asp305Asn	3.14×10^−05^	Asp	KEL:30	–	–
KEL*02.-32	Ala424Val	5.08×10^−06^	Ala	KEL:32	–	–
KEL*02.-33	Arg428Leu		Arg	KEL:33	–	–
KEL*02.-34	Tyr253Cys		Tyr	KEL:34	–	–
KEL*02.-35	Leu260PheArg675Gln		Leu+Arg	KEL:35	–	–

KEL*02.-36	Thr464Ile	2.47×10^−04^	Thr	KEL:36	–	–
KEL*02.39	Arg293Trp	1.53×10^−05^	**Arg**	KEL:37	**Trp**	KEL:39
KEL*02.31	Arg292Gln	1.09×10^−04^	**Arg**	KEL:38	**Gln**	KEL:31
KEL*02.41	Arg513Gln	2.12×10^−05^	**Arg**	KEL:40	**Gln**	KEL:41
**31**	**32**		**22**		**14**	

Residues in the wild-type Kell protein (ISBT allele KEL*02, “AgWT”) and single/double Kell mutations that can elicit an antibody response in individuals with a different KEL genotype (“AgV”) are indicated by the given amino acid in the respective columns. Alleles encoding antithetical antigens and respective amino acids are in boldface. Numbers in the last row show that a total of 32 missense variants are included. They lead to 22 different AgWT and 14 different AgV amino acids. Missense variants responsible for a total of 17 "minus" alleles are shaded in green. Frequencies refer to the MAF in the European population (gnomAD V4.1.0).

#### 2.1.2 Definition of control variants

Naturally occurring Kell variants with no recorded Kell phenotype or any detected antigenicity may be considered as non-antigenic and may well serve as negative controls for studying the mechanisms of Kell immunogenicity. To define a useful non-antigenic control group, only variants with a high enough occurrence in the population were considered: Kell variations observed extremely rarely or only in developing countries with poor medical care might have escaped detection of their antigenicity. Since there is variability in the allele frequencies of specific populations, we restricted our retrieval of control variant (denoted “CtrlV”) alleles to the European population. The gnomAD database (23), currently the most comprehensive human genome allele frequency reference dataset, lists 548 European Kell variations that have not yet been shown to be involved in Kell immunization (**Supplementary Table S2**). Most of these are very rare and therefore not suitable as negative controls. We chose a minor allele frequency (MAF) cutoff of 3.0×10^−05^, which is significantly higher than the MAF of several well-described antigenic Kell variants, assuming that any antigenicity, if it exists, would have already been detected in clinical practice until today. Applying this cutoff yielded 28 CtrlV from gnomAD. In addition, the AgWT dataset as defined in Section 2.1.1, includes 17 alleles, for which the variant amino acids have no proven antigenicity so far (therefore called 'minus' variants and alleles). Three of these variants meet our MAF cutoff of 3.0×10^−05^ and were therefore included in the CtrlV dataset (**Table 3**). The complete dataset comprising a total of total 101 Kell missense variants is listed in **Supplementary Table S1**.

**Table 3:** Control group of Kell variants of no known phenotype ranked by minor allele frequency (MAF) in the European population (gnomAD V4.1.0).

Non-antigenic Kell missense variants “CtrlV”	European MAF (cutoff 3.0×10^−05^)
Asp692Asn	5.02×10^−04^
Ile278Leu	4.58×10^−04^
Arg492Gln (KEL*02.-19)	3.44×10^−04^
Asp689Tyr	2.40×10^−04^
Tyr714Phe	2.13×10^−04^
Ala598Thr	1.42×10^−04^
Asp315Asn	1.41×10^−04^
Glu606Gln	1.03×10^−04^
Ala445Val	9.58×10^−05^
Thr421Met	9.49×10^−05^
Lys112Glu	9.41×10^−05^
His691Gln	9.26×10^−05^
Arg716His	8.98×10^−05^
Met156Val	7.20×10^−05^
Pro402Thr	6.87×10^−05^
Ile278Val	6.10×10^−05^
Ala645Thr	5.76×10^−05^
Ile242Met	5.68×10^−05^
Ala574Thr	5.68×10^−05^
Glu634Asp	4.83×10^−05^
Ala423Ser	4.32×10^−05^
Asn456Lys	4.23×10^−05^
Arg130Trp (KEL*02.-18.1)	4.15×10^−05^
Phe431Ser	4.14×10^−05^
Ala645Glu	3.47×10^−05^
Val340Met	3.39×10^−05^
Asp305Asn (KEL*02.-30)	3.14×10^−05^
Glu146Lys	3.30×10^−05^
Val268Leu	3.22×10^−05^
Glu413Lys	3.05×10^−05^
Ala712Thr	3.05×10^−05^
**Total number: 31**	

Only single amino acid exchange variants with a MAF ≥3.0×10^−05^ were included. Three variants from the AgWT dataset exceed the CtrlV cutoff and were therefore included in the CtrlV dataset (listed in bold type, with their ISBT allele name).

### 2.2 A structural model of the Kell protein

The protein structure of Kell has not yet been determined experimentally. We therefore constructed two *in silico* structural models: A full-length model by *ab initio* structure prediction of the Kell-Zn^2+^ metalloprotein generated by AlphaFold3 (24) and a partial model by comparative modeling of the extracellular domain only using Modeller (25), based on the known structures of two human paralogs as structural templates: human endothelin-converting enzyme I [ECE-1 (26)] and human neutral endopeptidase [NEP (27)], both from the M13 family of metallopeptidases and sharing a moderate sequence identity with Kell (31% and 24%, respectively). Membrane insertion borders of the full-length model were predicted by a molecular dynamics-based algorithm (PPM Server) (28). The AlphaFold3 model had a better overall geometry than the Modeller model and was also in good agreement with the experimentally determined homolog structures (**Supplementary Material Section 1**). It was therefore used as the primary model for all subsequent structure-based analyses; however, all findings were cross-validated against the Modeller model.

**Figure 1:**
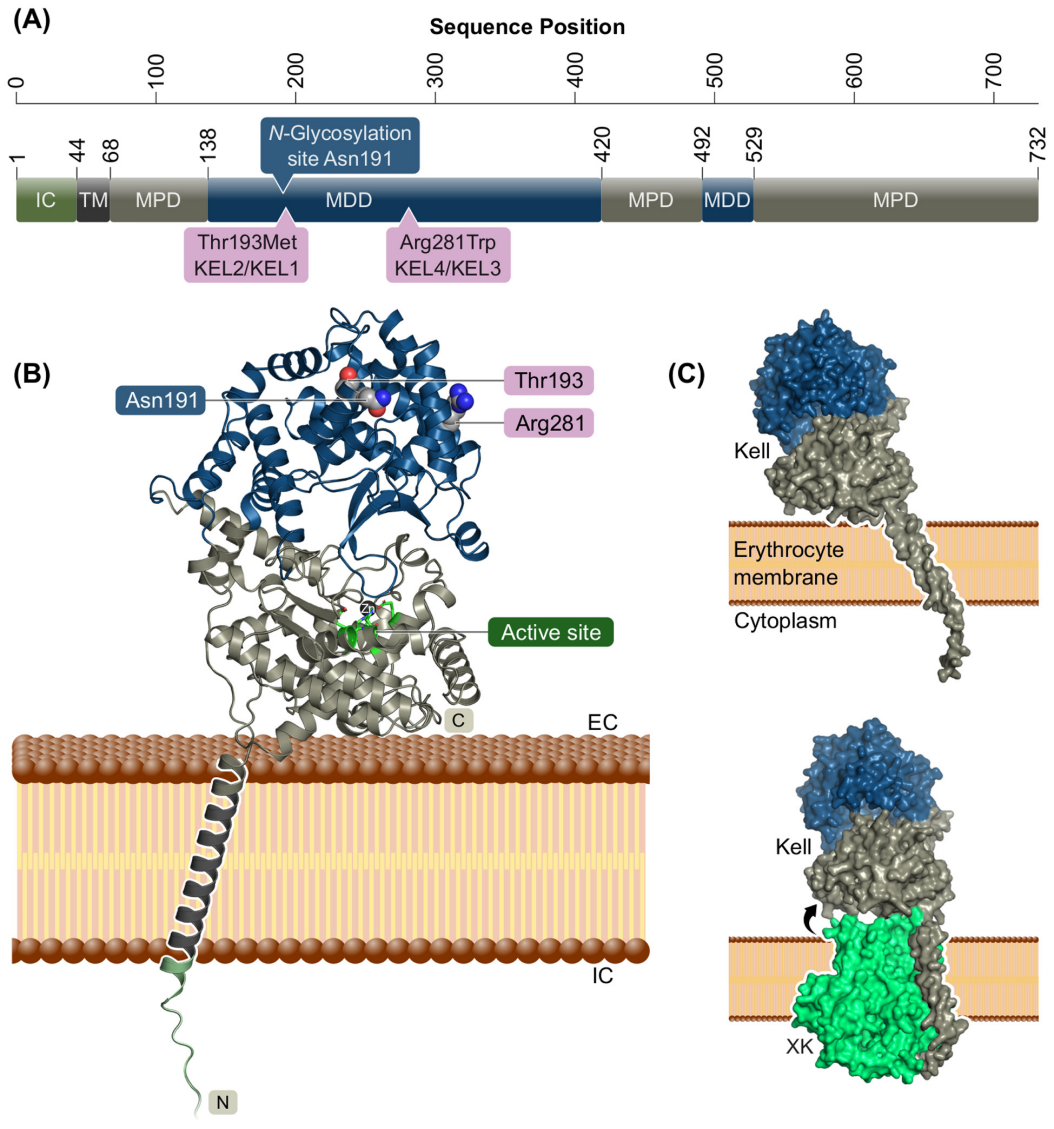
Kell protein domain composition and structural model. **(A)** Sequential organization of the Kell protein indicated by domain borders. IC, intracellular domain; TM, transmembrane helix; MPD, membrane-proximal domain; MDD, membrane-distal domain. **(B)** Protein structural model of Kell. MPD in gray, MDD in blue, active site in green stick representation with zinc atom as black sphere. The *N*-glycosylation site at Asn191 as well the wild-type residue of the two most common KEL variant sites Thr193Met (KEL2/KEL1) and Arg281Trp (KEL4/KEL3) are shown as spheres with carbon in gray, oxygen in red, and nitrogen in blue. Disordered intracellular N terminus omitted for clarity. **(C)** Predicted membrane orientation of AlphaFold3 models of Kell alone and in complex with XK. Binding to XK is predicted to induce a more membrane-perpendicular orientation of Kell.

The Kell protein is composed of a short cytoplasmic N-terminal region (residues 1–47), a single transmembrane α-helix (residues 48–67), and an extracellular domain (residues 68–732), the latter of which can be divided into two subdomains, a membrane-proximal domain (MPD) and a membrane-distal domain (MDD). The MPD and MDD are composed of non-consecutive sequential regions (**Figure 1A**) and form a two-lobed structure with a central substrate-binding cleft (**Figure 1B**). A part of the MPD surface is oriented towards the membrane surface. The MPD harbors the active site of the enzyme (Glu582, Asp638, Zn^2+^-binding His581, His585, and Glu634) and four out of five conserved disulfide bridges. The experimentally verified *N*-glycosylation site at Asn191 (11) is in an accessible position at the most membrane-distal surface of the MDD (**Figure 1B**). Since AlphaFold3 is able to predict protein–protein complexes, we also generated a model of Kell bound to its physiological binding partner XK (6). Interestingly, AlphaFold3 and PPM predict a slightly more perpendicular position of Kell relative to the membrane when in complex with XK (**Figure 1C**).

The structural model allows for the localization of all known Kell variants in 3D. Variant sites that are far in sequence may be structural neighbors and antigenic variant sites clustering on the surface may hint to physiologically relevant epitopes. Vicinity to the erythrocyte membrane and covalent binding of Kell to XK are likely to have an effect on the accessibility of epitopes (**Figures 1B, C**). The differences in observed expression levels of “NullV” versus “ModV” Kell mutations might also be rationalized in a context of protein folding and structure. We therefore set out to systematically analyze and compare all variants in our five pre-defined classes of Kell variants NullV, ModV, AgWT/AgV, and CtrlV, in order to find correlations between their structural context and mutational effect.

#### 2.2.1 Localization and conservation of *NullV* and *ModV* amino acid positions

**Figure 2:**
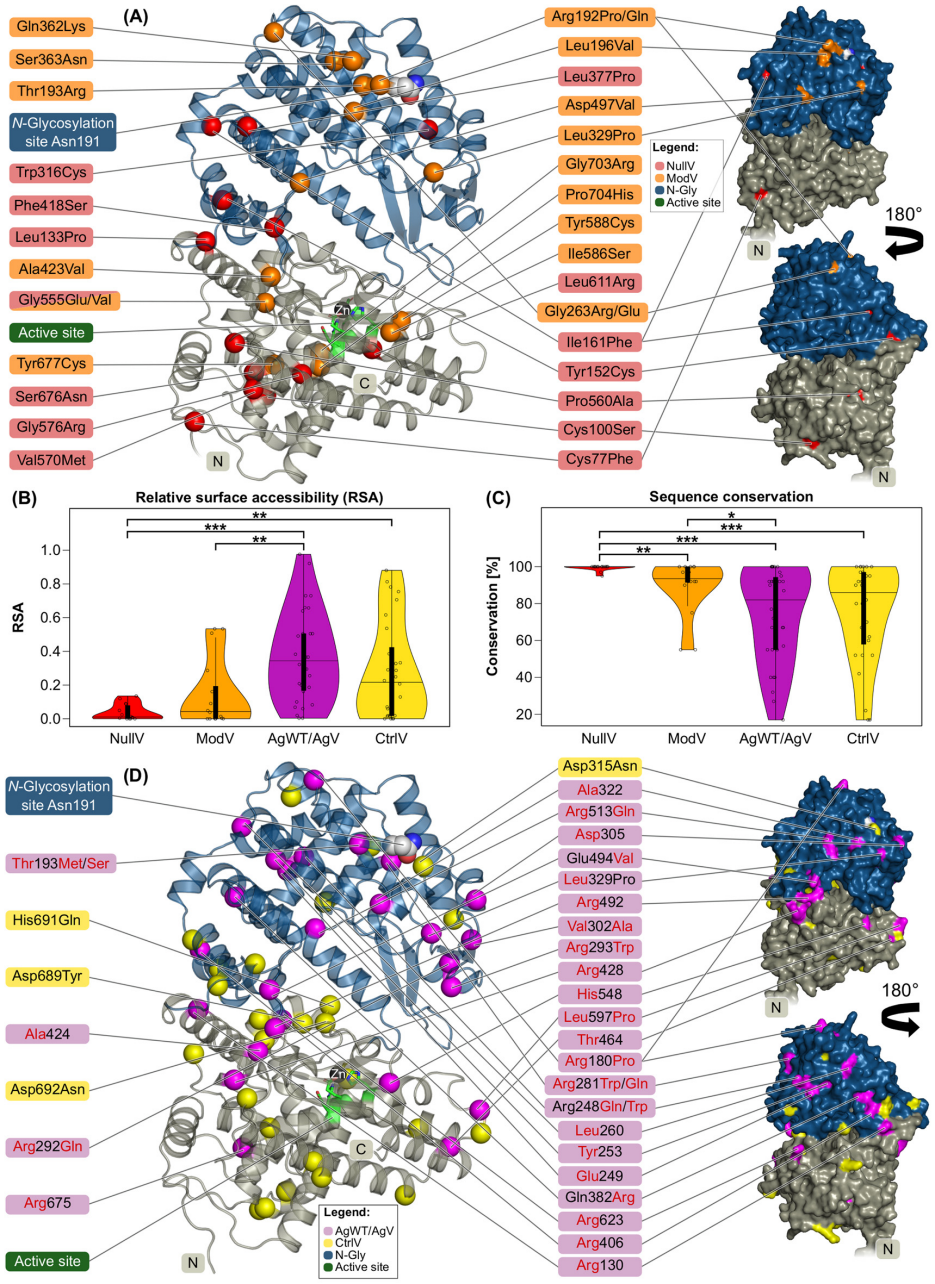
Localization, solvent accessibility and conservation of Kell protein variants. Domain coloring as in **Figure 1**. **(A)** Cartoon (left) and surface (right) representation of the Kell extracellular domain with NullV shown as Cα atom spheres in red and ModV in orange. **(B)** Distribution of relative solvent accessibility values of Kell variant residues. **(C)** Sequence conservation of Kell variant residues in percentage among 39 orthologs (alignment in **Supplementary File** Kell_Orthologs_Alignment.fasta). **(B, C)** Asterisks mark statistically significant differences between median values by asymptotic Wilcoxon–Mann–Whitney tests (*p ≤ 0.05, **p ≤ 0.01, ***p ≤ 0.001). The thick black bar in the center of the violin plot represents the interquartile range. **(D)** Cartoon (left) and surface (right) of the Kell extracellular domain with AgWT and AgV locations indicated as Cα atom spheres in magenta, and CtrlV in yellow. CtrlV mentioned in the main text are labeled. The surface is shown in the same orientation as the cartoon figure as well as rotated by 180°C.

The majority of NullV locate within the MPD, some of them near the active site, the others near the domain border to the MDD (**Figure 2A**). Interestingly, NullV cluster on one side of the molecule, which indicates that here the fold is less tolerant to variations than on the opposite side that harbors the enzymatic active site and surrounding flexible loops. Notably, the MPD is also structurally more conserved among Kell homologs than the MDD (**Supplementary Material Section 1**). All NullV positions are fully or partially buried with nearly no solvent accessibility [relative surface accessibility (RSA) values <0.15, **Figures 2A, B**]. Moreover, some NullV residues are in close vicinity of each other: Leu133 and Tyr152, Ile161 and Leu377, and Val570 and Ser676. Overall, NullV positions are sequentially and structurally strongly conserved among orthologs and paralogs (**Figure 2C**; for conservation among paralogs, see **Supplementary Figure S2**).

ModV mutations display a larger spread regarding both solvent accessibility and sequence conservation. Four variants near the C terminus locate to a structurally rigid region, are buried around the active site, and are therefore very likely to cause a structural destabilization: Ile586Ser (RSA = 0.03), Tyr588Cys (RSA = 0.17), Tyr677Cys (RSA = 0.01), and Gly703Arg (RSA = 0.0) (**Figure 2A**, **Table 4**). The other ModV are mostly clustering in the most membrane-distal region, exposed or near the surface, suggesting that mutations in this region destabilize the Kell fold locally, possibly preventing an efficient surface transport. One particularly interesting ModV is Thr193Arg, located at the position of the hallmark antigen Thr193Met (KEL1 or the K antigen). While neither of the antithetical variants (Thr193 or Met193) interfere with Kell expression levels, Arg193 causes a K_mod_ phenotype (**Table 1**). The large and positively charged arginine side chain in a buried location (RSA = 0.0) may cause folding issues compared to the non-polar methionine. Sequential and structural neighbors of Thr193 are also variant sites that cause K_mod_ phenotypes (Arg192Pro/Gln, Gln362Arg, and Ser363Asn) (**Figure 2A**). While a proline at position 192 may interfere with the α-helical secondary structure, a rationalization of the effects of the other mutations in this region is not straightforward.

**Table 4:** Relative solvent accessibilities (RSA) of selected Kell residues discussed in the text.

Kell Missense variants	KEL Variant class	RSA AlphaFold3	RSAModeller
Leu133Pro	NullV	0.025	0.007
Tyr152Cys	NullV	0.010	0.010
Ile161Phe	NullV	0.054	0.030
Arg180Cys/His/Pro	AgWT/AgV	0.515	0.081
Arg192Pro/Gln	ModV	0.535	0.419
Thr193Met/Ser/Arg	AgWT/AgV/ModV	0.000	0.001
Leu260Phe	AgWT	0.244	0.153
Arg281Trp/Gln	AgWT/AgV	0.351	0.301
Gln362Lys	ModV	0.026	0.011
Leu377Pro	NullV	0.026	0.025
Val570Met	NullV	0.000	0.019
Ile586Ser	ModV	0.030	0.003
Tyr588Cys	ModV	0.171	0.177
Leu597Pro	AgWT/AgV	0.995	0.242
Arg675Gln	AgWT	0.040	0.045
Ser676Asn	NullV	0.002	0.006
Tyr677Cys	ModV	0.010	0.036
Asp689Tyr	CtrlV	0.567	0.357
His691Gln	CtrlV	0.772	0.516
Asp692Asn	CtrlV	0.398	0.583
Gly703Arg	ModV	0.000	0.038
Buried: 0.00 ≤ RSA < 0.05				
Half-buried: 0.05 ≤ RSA < 0.25				
Exposed: 0.25 ≤ RSA < 1				

Values are given for wild-type amino acids in the context of the structural model produced by AlphaFold3 and Modeller for cross-validation. Background colors as indicated in the bottom highlight buried, half-buried and exposed amino acids.

In summary, NullV and ModV positions are clearly the most conserved of all Kell variants and localize primarily in positions with no or very low solvent accessibility. NullV are more conserved and less solvent accessible than ModV (**Figures 2B, C**). Sequential and structural conservations and the RSA of every variant amino acid position are listed in **Supplementary Table S3**.

#### 2.2.2 Localization and conservation of AgWT/AgV amino acid positions

AgWT/AgV are not evenly distributed across the Kell molecule surface: the membrane-facing side and the most membrane-proximal part of the MPD are completely spared from AgWT/AgV, and some surface regions at the very top and on one side of the MDD show less antigenic variants than the rest (**Figure 2D**). Some antigenic positions are fully buried (RSA < 0.05) or half-buried (RSA between 0.05 and 0.25) (**Table 4**). The most exposed top region harbors the *N*-glycosylation site with Asn191-Arg192-Thr193 containing the most prevalent antigenic site, Thr193 in wild-type (KEL2) or Met193 in the most common variant. Thr193 (RSA = 0.0) is sandwiched between surface helices and the preceding loop 177–189, suggesting that the substitution modifies the conformation of the very top of the molecule without destabilizing the fold, but by preventing *N*-glycosylation. Interestingly, this loop also includes AgWT/AgV position Arg180 (RSA = 0.51). Substitution of Arg180 with proline results in an antithetical pair (KEL14/KEL24, **Table 2**). However, individuals with Arg180Cys or Arg180His carry a non-antigenic “minus” allele and can raise antibodies against the wild-type Arg180. Likely, substitution of Arg180 changes the properties of the surface loop, and it remains to be tested whether the mutation interferes with *N*-glycosylation. The second most clinically relevant antigenic site, Arg281Trp (KEL3), is fairly exposed at the side of the molecule (RSA = 0.37) and involves a loss of positive surface charge due to the arginine mutating to uncharged tryptophan. The variant contributing to the KEL35 antigen, Arg675, is deeply buried (RSA = 0.04) in the MPD. The second residue in KEL35, Leu260, however, locates more exposed (RSA = 0.24) at the MDD, confirming an earlier suggestion that it is the determining factor for the KEL35 phenotype (18) (**Figures 2B, D**, **Table 2**).

Non-antigenic missense variants (CtrlV, **Table 3**), in contrast to AgWT/AgV, are distributed almost equally throughout the Kell extracellular domain (**Figure 2D**) and appear in both buried and exposed locations. Some CtrlV are spatial or sequential neighbors of AgV. While many arginine residues are found in the AgWT/AgV dataset, only a single arginine substitution is included in the CtrlV group (Arg716His), locating among many other CtrlV in the MPD, suggesting that the region is less accessible for recognition by immune receptors due to its vicinity to either the membrane surface, in line with earlier suggestions (18), or the XK protein (**Figure 1C**). Very few CtrlV positions are strongly conserved in sequence and structure (**Figure 2C**, **Supplementary Table S3**). If they are deeply buried, they locate at a large central cavity, a less densely packed region in the structural core that seems to be more tolerant to mutations than other regions. The most common CtrlV, Asp692Asn, locates in this cavity, as well as Asp689Tyr and His691Gln (**Figure 2D**).

To facilitate the analysis of variant positions, a PyMOL session file is provided that enables to view variant amino acid positions in 3D (**Supplementary File** KEL_Pymolscript.pse included in the Supplementary folder Structural Modeling).

### 2.3 Physicochemical properties of variant sites

Every amino acid is unique in its properties that determine the structural and functional role of the residue within a protein structure. Besides hydrophobicity, which determines the preference of an amino acid to be in a buried location, other properties may be important, such as side chain volume and flexibility. Variant amino acids that cause steric clashes in a densely packed environment can destabilize the protein locally or globally. It is therefore commonly assumed that amino acid substitutions are less likely to have an influence on expression and function of the protein if the properties of wild-type and variant amino acid side chains are similar. Numerical values for diverse amino acid properties determined by experimental and statistical methods were retrieved from the AAIndex database (29, 30) (**Supplementary Material Section 12**) and compared.

#### 2.3.1 Comparison of variations among datasets NullV, ModV, AgWT/AgV, and CtrlV

**Figure 3:**
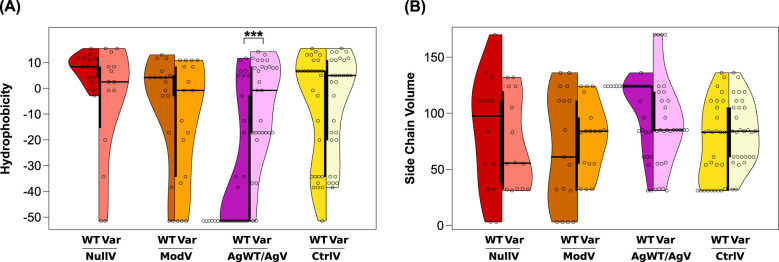
**(A)** Hydrophobicity and **(B)** side chain volumes of amino acids in variant sites of the Kell protein. Distribution of property scores of the wild-type amino acids on the left side of each violin plot and variant amino acid scores on the right side. Asterisks mark statistically significant differences between median values by asymptotic Wilcoxon–Mann–Whitney tests (***p ≤ 0.001). The thick black bar in the center of the violin plot represents the interquartile range.

We chose to compare the numerical values for hydrophobicity and side chain volume of all wild-type and variant amino acids collected in our dataset (**Supplementary Table S3**). The distribution of scores among the wild-type positions of 14 NullV, 17 ModV (**Table 1**), 32 AgWT/AgV (**Table 2**), and 31 CtrlV (**Table 3**) sites, compared to their respective substitutions, is shown in **Figure 3** and **Supplementary Material Section 3**).

Panels A and B in **Figure 3** illustrate the comparison between wild-type and variant values in the different datasets. The plots visualize characteristic value ranges for all dataset groups and differences in both the spread and the median of values, apparent from an asymmetry in the split violin plots of NullV, ModV, and AgWT/AgV. In contrast, plots of the CtrlV group are nearly symmetrical. Wild-type residues at NullV and ModV positions are more hydrophobic than wild-type residues at AgWT/AgV positions (**Figure 3A**), as would be expected for mostly buried and mostly surface-exposed sites, respectively. After substitution, the median hydrophobicity of NullV residues decreases, and the spread of values increases, including some highly hydrophilic substitutions (**Figure 3A**). An opposite trend is seen for AgWT/AgV substitutions, clearly due to the many occurrences where very hydrophilic arginine side chains are substituted (**Table 2**). In fact, the median value of AgWT/AgV variants approaches that of the CtrlV. CtrlV substitutions show the least changes of properties, in line with them being mostly conservative mutations without any disruptive effects or introduction of antigenicity (**Figure 3A**).

The side chain volumes of wild-type residues are more widely spread in NullV and ModV than in AgWT/AgV and CtrlV (**Figure 3B**). NullV substitutions, interestingly, tend to involve a reduction in side chain volume, as do AgWT/AgV substitutions, again the latter being dominated by numerous arginine substitutions to smaller side chains (**Figure 3B**). Since NullV substitutions are typically located in densely packed buried positions within the protein, smaller side chains are likely to reduce structural cohesion leading to the K_0_ phenotype. A similar but less obvious trend is observed for ModV.

More plots highlighting the difference between wild-type and variant positions are provided in **Supplementary Material Section 3**. Further analyses conducted to view the differences between the Kell variant classes include protein structural destabilization predictions and several functional predictions and annotations that were obtained from the dbNSFP metaserver (**Supplementary Material Sections 4** and **5**, **Supplementary Table S3**, and collection of R plots in **Supplementary Data file** dbNSFP_Violinplots.zip).

#### 2.3.2 Comparison of biophysical properties between antigenic and non-antigenic amino acids

**Figure 4:**
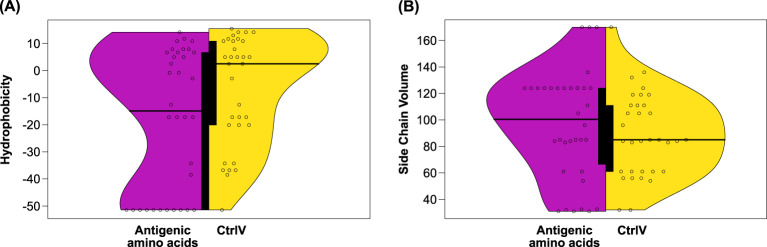
**(A)** Hydrophobicity and **(B)** side chain volumes of antigenic vs. non-antigenic amino acids of the Kell protein. The distribution of property scores among antigenic amino acids of the AgWT/AgV dataset (magenta) was compared to properties of the variant amino acids of the non-antigenic CtrlV dataset (yellow). Differences in mean hydrophobicity were near statistical significance (*p* = 0.06), whereas side chain volume differences were not statistically significant (*p* = 0.24). The thick black bar in the center of the violin plot represents the interquartile range.

Following the comparison of overall characteristics of amino acid substitutions in Kell protein variants, we were interested whether some properties are characteristic for the antigenicity of a given amino acid. We collected amino acid properties for 22 AgWT and 14 AgV residues (**Table 2**) and variant residues from 31 non-antigenic CtrlV (**Table 3**) and compared the properties among the two subsets. As shown in **Figure 4**, AgWT/AgV amino acids are generally less hydrophobic and have larger side chains than CtrlV. Despite their higher hydrophilicity compared to CtrlV, they are not more solvent accessible (**Figure 2A**). It is important to note that 50% (11 out of 22) of AgWT residues are positively charged arginine residues, and although arginine is very hydrophilic, many of these are partially buried (**Supplementary Table S4**).

In conclusion, two different mutational consequences lead to the establishment of an antigen: (i) the loss of arginine on the surface supports the theory that a change of surface charge is an important factor determining immunogenicity; (ii) the substitution of buried amino acids can indirectly lead to the creation of a surface antigen by causing local structural changes. This can happen when the physicochemical properties of the substituted amino acid impact a conformationally sensitive region such as sites of interaction between secondary structure elements.

### 2.4 Prediction of conformational epitopes

A non-self extracellular protein is recognized by antibodies in specific regions that are referred to as epitopes. On the surface of the folded protein, those regions can be composed of sequentially discontinuous residues that co-localize in 3D—so-called conformational epitopes.

Automated prediction methods of conformational epitopes are still very limited due to missing structural data of known antigens (31). Hence, *in silico* epitope prediction methods are not able to specifically identify known Kell antigens. Discotope (32), BepiPred (33), and others predict randomly distributed sites (**Supplementary Material Sections 6** and **7**). A visual inspection of the antigen locations in the 3D structural model of the Kell protein showed that many AgWT/AgV co-localize in 3D on the same or adjacent secondary structural elements. Therefore, we predicted several epitopes, based on the suggestion that exposed and especially half-buried antigenic amino acid substitutions are not critical for the global protein structural fold but can create a conformational change on the surface that can be recognized by the immune system of the recipient of a blood transfusion.

**Figure 5:**
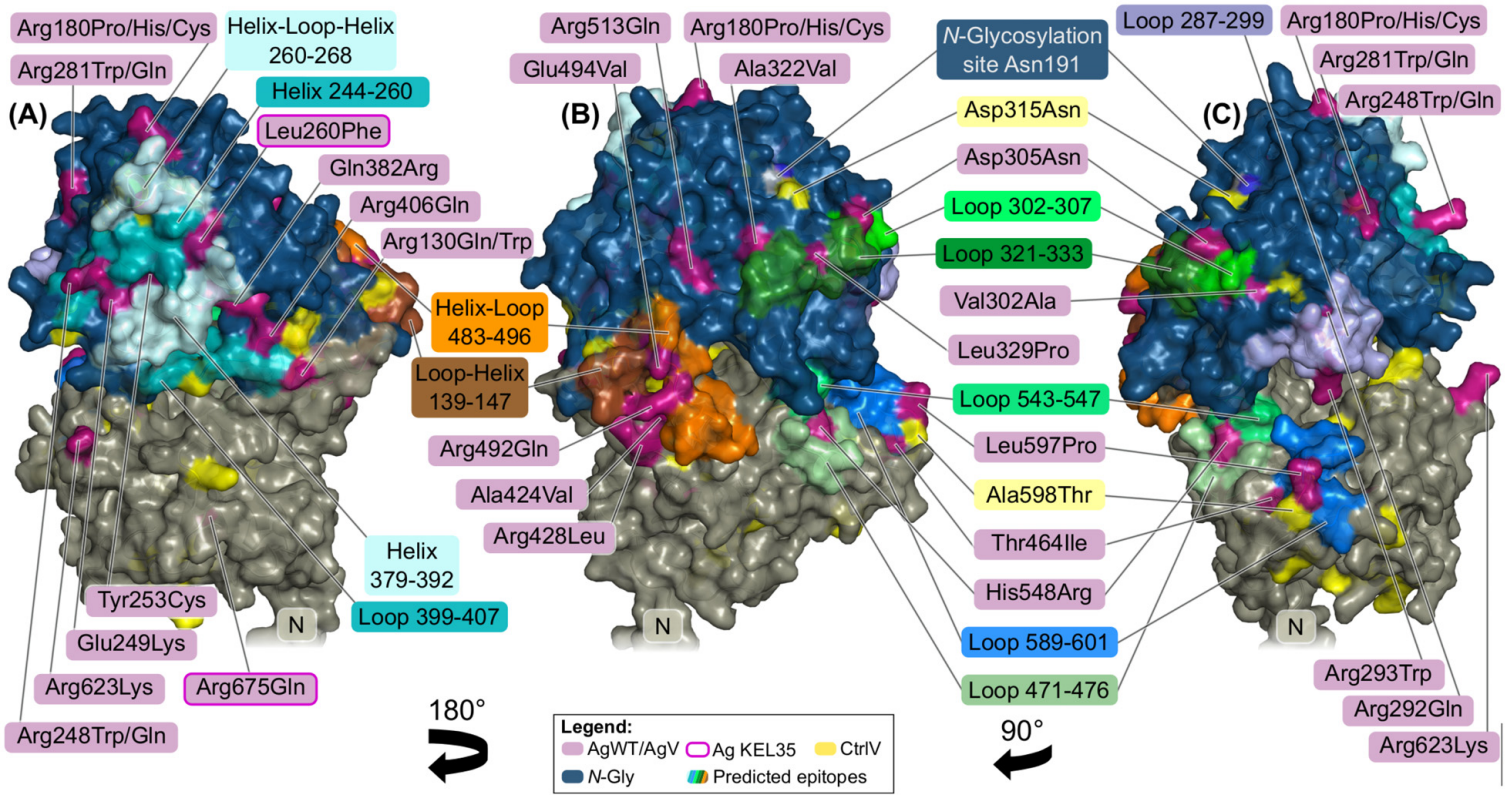
Predicted conformational epitopes on the surface of the predicted Kell protein structure. Different colors indicate different secondary structure elements composing the epitopes. **(A)** An epitope (cyan) ranging over two helices and possibly including adjacent loops. Individual contributing residues are labeled in magenta. Two AgWT defining the KEL35 antigen are highlighted with pink outlines. Leu260 is part of the predicted epitope, possibly affecting a surface loop when mutated to Phe, while Arg675Gln is buried. **(B)** Ala424Val and Glu494Val likely destabilize the helix–loop 483–496 (orange), while, in contrast, Arg428Leu and Arg492Gln only change surface charge. AgWT Asp305 and Ala322 are accessible and contribute to a predicted epitope (green) together with half-buried AgWT Leu329. **(C)** A predicted epitope (blue) with centrally locating Leu597Pro (KEL6/KEL7) inside a loop (589–601). Nearby is another long loop (287–299) that harbors two antithetical antigens: exposed Arg293Trp and buried Arg292Gln.

In the structural model, AgWT positions Glu249, Tyr253, and Leu260 are neighbors on the same helix (position 244–260), together with AgV Arg248Gln and Arg248Trp. Arg248 is fully exposed to the solvent (RSA = 0.73), suggesting a direct antigenic effect by the loss of positive charge on the protein surface (**Figures 5A, C**). The other variants, in contrast, are half or fully buried. The three AgWT amino acids are all highly conserved, and in our model, they contact the adjacent helix (position 379–392), making it a central part of the predicted epitope. The model suggests that these variations could induce a local structural change by disturbing intramolecular interactions between the two helices. The substitution Leu260Phe may also alter the consecutive surface helix–loop–helix structure (position 260–268).

The adjacent helix 379–392 harbors AgV Gln382Arg that is part of another predicted epitope. Gln382 is also half-buried (RSA = 0.16) and in our model is in interaction distance with AgWT Arg406, thereby connecting the helix with the neighboring loop (399–407) (**Figure 5A**).

On the other side of the MDD, AgWT Asp305 and the antithetical variation site Val302Ala are on either end of the same strand–loop–helix structure (position 300–310) and are therefore possibly part of the same conformational epitope. This region is in contact with loop 322–333, whose conformation may also be influenced by AgWT Ala322 (**Figures 5B, C**) or AgWT/ModV Leu329Pro.

A structural cluster in our model is also formed by AgWT Ala424 and Arg428, together with AgWT Arg492 and AgV Glu494Val (**Figure 5B**). Both arginine sites are very exposed, and their mutation leads to loss of antigenicity. Ala424 and partly Glu494, in contrast, are buried and may therefore define the 3D conformation of helix–loop–helix 483–496, leading to a conformational antigen, possibly together with the neighboring loop–helix structure 139–147 (**Figure 5B**).

Two sequentially adjacent buried antithetical variants Arg292Gln and Arg293Trp are predicted to create a change of the loop (position 287–299) on which they are located (**Figure 5C**). In our model, Arg292 would be able to form a buried salt bridge with Asp226 and could therefore stabilize the loop at its position. Nearby, a long loop (589–601) harbors the antithetical antigens Leu597Pro (KEL6/7). Interestingly, Pro597, which is known to induce a strong immune response, is predominant in Kell orthologs (**Supplementary File** Kell_Orthologs_Alignment.fasta) and has a very high incidence in the African population with almost 9%, while the MAF in Caucasians is only 0.04%.

In conclusion, our model suggests that most Kell antigens are defined by a local conformational arrangement of surface helices and adjacent loops, where changes by mutation can lead to alternative surface shapes without disrupting the protein fold. Kell sequential regions comprising the predicted epitopes are indicated in **Supplementary Figure S11**.

Interestingly, more than half of the antigenic variations include arginine and glutamine, suggesting that these amino acid side chains are important for recognition by immune receptors. Six very exposed AgWT/AgV arginine residues locate on surface helices (R130, R248, R281, R428, R492, and R513) that are not flexible in their position or shape, assuming that the gain or the loss of a positive surface charge is sufficient in these cases. However, surface charge changes may also be the result of restructured loops and could translocate previously (partially) buried arginine side chains to the surface. RSA for all AgWT/AgV are listed in **Supplementary Table S4**.

### 2.5 HLA binding predictions of variant Kell peptides

For a robust antibody production, B cells need to receive a co-stimulatory signal from CD4^+^ T cells, usually in the form of cytokines secreted after recognition of a cognate peptide–HLA-II complex on the surface of B cells. HLA-II proteins present linear peptides that are between 13 and 17 amino acids long. The genes encoding HLA-II proteins, namely, HLA-[DRA, DRB, DQA, DQB, DPA, DPB], are highly polymorphic. To investigate the impact of HLA-genetic variability on the presentation of peptides encoded by different KEL alleles, 15-mer peptides with Kell antigenic variants (AgWT/AgV) as well as non-antigenic Kell variants (CtrlV) were submitted to PIA (34) to computationally estimate their presentation probability, i.e., their likelihood of presentation by the corresponding HLA-DRB1 alleles. Different trends were observed across the 19 HLA-DRB1 alleles tested; however, no significant difference between the different groups was observed among the test alleles (**Supplementary Figure S9**).

### 2.6 An applicable strategy to annotate new Kell blood group antigens

Based on the insights gained in this study, we compiled a list of specific conditions that represent typical properties of destabilizing NullV/ModV and antigenic AgWT/AgV positions (**Supplementary Material Section 10**) that could be queried by serologists if encountered with new Kell variants.

We applied this strategy to 19,772 exomes that were obtained from an in-house whole exome sequencing dataset. It included 1,486 KEL1 and 318 KEL3 heterozygous genotypes, and 35 KEL1 and 3 KEL3 homozygous individuals. A total of 140 exomes were identified as carrying other AgWT/AgV alleles and additional 15 exomes included a known K_0_ or K_mod_ allele.

In total, 128 different Kell missense variants were detected, of which 46 were known blood group variants and CtrlV that were already included in our dataset. A total of 61 variants were listed in the gnomAD with a MAF smaller than our control cutoff of 3.0×10^−05^ and additional 21 new Kell variants were found (**Supplementary Table S6**). The 82 as-yet-uncharacterized variants were subjected to the following annotation strategy:

Guideline for the annotation of new Kell variants:

1. Variant at known antigenic (AgWT/AgV) or destabilizing (NullV/ModV) Kell position.
2. Sequentially conserved in 39 Kell orthologs.
3. Structurally conserved in at least one of the three M13 family members with known protein structures.
4. Solvent accessible or buried in combination with at least one of the following physicochemical property changes:
  a. Amino acid substitution from or to proline.
  b. Change of side chain volume.
  c. Change of charge on the surface.
  d. From hydrophobic amino acid side chain to hydrophilic in a buried position.
  e. From hydrophilic amino acid side chain to hydrophobic at an exposed position.
5. Substitution includes arginine, glutamine, tryptophan, or proline.
6. Location in the 3D structural model:
  a. Not located at an inside cavity or membrane-proximal.
  b. Within predicted epitopes (**Figure 5**).
7. An MPC score above 0.4. (e.g., from the dbNSFP server).

Application of the list of conditions to detect potential new K_0_ variants or KEL antigens resulted in the selection of 19 Kell variants (**Supplementary Material Section 10** and **Supplementary Table S6**). An example of a conditional path for predicting the phenotype of a variant is given in **Supplementary Figure S10-1**. Interestingly, in the Kell structural model, a similar 3D distribution compared with the Kell dataset variants of the NullV/ModV and AgWT/AgV categories was observed (**Supplementary Figure S10-2**). Four rare mutations identified from the Erythrogene database in a previous Kell variant study (18) were also analyzed, and indeed, two of them (Pro621Arg and Met479Ile) were predicted to have antigenic properties (**Supplementary Table S6**).

The exome sequence database used herein was not originally set up to serve for this purpose, and individuals found to be carrying known or predicted Kell destabilizing or antigenic variants cannot be followed up to test them experimentally at this point. However, for present or future blood transfusion databases, this approach to detect potentially immunogenic gene variants may be worthwhile to explore.

## 3 Discussion

This study provides the first comprehensive approach to deriving the molecular determinants of Kell immunogenicity. The Kell blood group system poses an excellent model to systematically analyze missense variations: Firstly, NullV and ModV affect very conserved, mostly buried locations and the substitutions likely destabilize the fold. Second, AgWT/AgV typically affect surface changes, and third, CtrlV do not affect protein stability and are not immunogenic, as we conclude from their high frequency in the European population.

The analyses revealed clear trends of localization, conservation, and amino acid properties of variant sites classified in the four groups, NullV, ModV, AgWT/AgV, and CtrlV, and showed characteristic differences in amino acid properties comparing antigenic and non-antigenic sites. A buried localization of the substituted amino acid combined with a strong evolutionary conservation, side chain volume, and hydrophobicity changes added to the K_0_ and K_mod_ phenotype, and most variations involving charge on the surface lead to the creation of antigens. Controls, i.e., commonly occurring non-antigenic, non-destabilizing Kell variants, showed clearly less structural and physicochemical changes upon substitution. Nevertheless, the tolerance of some CtrlV mutations is surprising from a structural point of view, especially if they are buried and in close vicinity of NullV or ModV. Possibly, some specific amino acids are generally less immunogenic; for example, threonine, methionine, lysine, and asparagine are not typically observed in antigenic positions (AgWT and AgV), but each of them is more than fivefold more common in in the CtrlV dataset than in the overall Kell sequence. On the other hand, arginine, glutamine and tryptophan residues are overrepresented in the AgWT/AgV group (**Supplementary Figure S8**).

The 3D distribution of variants when plotted on our Kell structural model provided interesting insight into the structural characteristics of the Kell protein. While the exact position of individual residues needs to be viewed with caution due to their dependence on the accuracy of the model, some overall trends become clear and are supported by both independent Kell models and experimental structures from homologous proteins. Destabilizing NullV are clustering in some regions pointing to structural rigidity, while the toleration of buried and even very common CtrlV indicated a loosely packed central cavity that appears flexible enough to accommodate amino acid substitutions. The lack of antigenic variant sites in the MPD indicates that it might be less accessible by antibodies. Perhaps, the *N*-glycosylation site on the MDD is also shielding the nearby surface from antibodies in a KEL2 background. Interestingly, one side of our Kell model is flatter and includes less variations than the opposite side, inviting speculations about a putative protein–protein interaction site.

Using the structural model, we predicted several structural epitopes on the protein surface (**Figure 5**). Many antigenic variant sites are half buried in the model of the wild-type protein, and since these substitutions do not interfere with Kell expression levels, we conclude that the introduced antigenicity must be the consequence of local structural adaptations leading to surface alterations. In fact, we observed that many AgWT and AgV co-localize in 3D and affect the same surface structures, such as loops or connection between adjacent helices. Very exposed AgWT/AgV are often modifying the charge, which is apparently sufficient to be sensed by immune receptors.

A high-resolution protein structure obtained by x-ray crystallography would provide a better basis to study potentially antigenic surface conformations, and molecular dynamics simulations could allow for further structural analyses such as accessibility in a membrane context and local flexibility changes following amino acid substitutions. The lack of significant differences between the AgWT/AgV and CtrlV groups regarding their predicted presentation by different HLA proteins reflects the complexity of the immune system and how it can distinguish self from non-self. Although presentation by HLA proteins is necessary for a T cell-mediated immune-response, it is not sufficient to drive an immune response: an antigen-specific T cell must recognize the presented peptide as non-self and mount an immune response toward this antigen. Different factors affect the ability of T cells to recognize a peptide as non-self, such as (i) the sequence of the T-cell receptor, which results from positive and negative selection in the thymus; (ii) the similarity of the peptide to other self-peptides; and (iii) signaling through other receptors such as pattern recognition receptors. In addition, our analysis of peptide–HLA interactions used peptide sequences without considering any possible post-translational modifications that could affect the interaction between peptides and HLA-II proteins or the upstream processes of enzymatic digestion and loading onto HLA-II proteins.

Finally, we predicted potential new antigens and destabilizing mutations in KEL sequences derived from 19,772 exomes from an in-house exome sequencing project. The observations gained throughout this analysis were used to create a conditional filter for amino acid substitutions that may indicate if a new Kell variant has the potential to destabilize the protein or alternatively to create an antigen on the protein surface. Applying this filter, we identified 19 variants, of which half are predicted to be destabilizing. The filter presented here can be easily applied by serologists for an initial evaluation of novel Kell variants with potential medical implications.

## 4 Methods

### 4.1 Dataset collection

All missense variants included in the ISBT (International Society of Blood Transfusion) database (9) were extracted from the KEL data sheet, excluding mutations that change the length of the *KEL* transcript by introducing a premature stop codon. Control missense variants were collected from the gnomADv4.1.0 (23) above MAF 3.0×10^−05^ in the European population. All missense variants included in this study are listed in **Supplementary Table S1**.

### 4.2 Protein sequence analysis

Kell ortholog sequences were obtained from the Ensembl database and aligned using Muscle (35). Incomplete sequences were excluded, resulting in an alignment of 40 sequences (**Supplementary File** Kell_Orthologs_Alignment.fasta). Human Kell Ensembl IDs are as follows: ENSP00000347409.2, transcript ENST00000355265.7g, and gene ENSG00000197993. Paralog sequences used for comparative modeling were obtained from the PDB. They are members of the M13 family of metallopeptidases (Conserved Domain Database ID cd08662) (36).

### 4.3 Generation of a Kell structural model

The protein structural model of full-length Kell and of the Kell extracellular domain (sequence position 75–732, from UniProt accession P23276) (4) were constructed with AlphaFold3 (24) and Modeller (37), respectively. The latter was given two template structures to increase model reliability. Template 1: PDB ID 3dwb, chain A (26), Human ECE-1 (endothelin-converting enzyme I), 31% sequence identity. Template 2: PDB ID 1dmt, chain A (27), Human NEP (neutral endopeptidase), 24% sequence identity. The two templates are 38% sequence identical and the only experimentally solved M13 family members that conserved the same stabilizing disulfide bridges and functional site residues as Kell. Both template structures were crystallized in complex with the metalloproteinase inhibitor phosphoramidon, and therefore show the enzyme in the closed conformation. The extracellular parts of the models were checked for geometry and compared to each other and the templates for validation (**Supplementary Material Section 1**). Only AlphaFold3 delivers predictions of transmembrane and intracellular segments (38) due to the lack of respective templates for comparative modeling. The position of membrane boundaries was predicted with the PPM server (39). Variant amino acid positions were visualized in the model using PyMOL (40). Amino acid residue surface accessibility for every variant position was provided by Discotope (33), which was also used to predict conformational epitopes from the structural model. Prior to construction of the input multiple sequence alignment for Modeller, the structural templates were aligned in 3D using FatCat (41) to verify structurally corresponding amino acid positions.

**Supplementary File** Structural_Modelling.zip includes (1) the AlphaFold3 model and PyMOL session file KEL_Pymolscript.pse with selected positions and (2) the Modeller model including Python2 script, template-target alignment, and structural template files.

### 4.4 Localization and conservation of Kell variants

In order to investigate the conservation of individual Kell amino acid positions, both sequence and structural alignments of homologous Kell proteins were analyzed. Conservation scores (0%–100%) of human Kell compared to 39 other mammalian Kell sequences (alignment in **Supplementary File** Kell_Orthologs_Alignment.fasta) are listed in **Supplementary Table S3**. To estimate the structural conservation of individual Kell amino acid positions, we compared our Kell model with the three available known structures of related proteins: the two remote human paralogs used for structural modeling, ECE-1 and NEP, and the bacterial protein Zmp1, a remote homolog from *Mycobacterium tuberculosis* (PDB ID 3ZUK) (42), a bacterial member of the zinc-dependent M13 endopeptidase family. Kell and Zmp1 share only 20% sequence identity and Zmp1 lacks the disulfide bonds conserved in Kell, NEP and ECE-1. Nevertheless, Zmp1 shares the same fold and enzymatic function, and comparing remote homologs on the structural level improves the reliability of the structural model. Structural conservation scores (values up to 3, if conserved in one, two, or all three structures) of all variant Kell amino acid positions are listed in **Supplementary Table S3**. The protein structural model of Kell was analyzed regarding the following: The spatial localization of Kell dataset missense variants regarding distribution among the membrane proximal and membrane distal subdomains, their surface accessibility, 3D clustering of variant types, structural–sequential conservation and predicted intramolecular interactions, and surface accessibility was determined in order to assess the mutational impact. FatCat (41) and POSA (43) were used for pairwise and multiple structural alignments, respectively.

### 4.5 Property analyses of variant sites

Numerical values for physicochemical properties were downloaded from the AAIndex database (29,30): hydrophobicity (PRAM900101), polarity (GRAR740102) and side chain molecular volume (GRAR740103) (**Supplementary Material Section 12**). For more visual clarity in the R plots showing the distribution of values, an inverted value of the hydrophobicity score was used, because stronger hydrophobicity is usually presented by lower numbers (kJ/mol). A delta *Z*-score was calculated for every substitution:


$$\Delta Z=\frac{Score_{Variant}-\bar{x}}{\sigma }\;-\;\frac{Score_{Wildtype}-\bar{x}}{\sigma }$$

where $\bar{x}$ refers to the mean and σ refers to the standard deviation. Violin plots to visualize the distribution of the obtained values with respect to their variant class were generated with R.

### 4.6 Prediction of variant structural and functional effect

We predicted the effect of Kell variants on protein structural stability using MAESTROweb (44) and RaSP (45). The two methods provide predicted stability changes for all possible amino acid substitutions by ΔΔ*G* scores, which is the difference of the Gibbs free energy of unfolding Δ*G* between the mutated and the wild-type protein structures.⁠ Distributions of ΔΔ*G* scores of all variant protein structures were visualized with violin plots by R. We also submitted all dataset missense variations to the dbNSFP webserver (46) (**Supplementary Table S7**) and visualized the distribution of scores with R violin plots (**Supplementary File** dbNSFP_Violinplots.zip).

### 4.7 Epitope predictions

The prediction of conformational epitopes from the protein structural model of Kell was supported by using Discotope (32) (https://services.healthtech.dtu.dk/services/DiscoTope-3.0), and linear epitopes were predicted from the protein sequence using BepiPred (33) (https://services.healthtech.dtu.dk/services/BepiPred-3.0/).

### 4.8 HLA binding predictions

HLA binding predictions were conducted using PIA-S/M by submission of 15-amino-acid peptides with AgWT/AgV and CtrlV on every possible position to the IKMB Hybrid Computing Service (https://hybridcomputing.ikmb.uni-kiel.de/) (34). Scores above the threshold 0.9 were considered significant.

### 4.9 Sequencing and secondary analysis of data

Library preparation was conducted from 500 ng of genomic DNA by Regeneron Pharmaceuticals using the NEBNext Ultra II FS DNA library prep kit (New England Biolabs). Whole exome hybridization enrichment was conducted using a modified xGen exome probe panel (Integrated DNA Technologies). Sequencing was done on a NovaSeq 6000 platform using a 2 × 75 bp S2 flow cell (both Illumina). Raw reads were converted to FASTQ files and reads were assigned to their specific barcodes. Alignment to GRCh38 was performed with BWA-mem (47). Duplicates were determined by Picard tools (48). Genome-wide SNVs and INDELs were called from the alignment files using WeCall (49). Variant effects were predicted and annotated using VEP (50). For further details and protocol modifications, please refer to (51).

### 4.10 Samples

The samples sequenced by Regeneron Pharmaceuticals stem from several biobanks of the University Medical Center Schleswig-Holstein, namely, the PopGen biobank, the Institute of Transfusion Medicine biobank, and the Clinic of Dermatology, Venereology and Allergology biobank. A total of 19,772 samples have been analyzed for their Kell variations.

## Data Availability

Exome sequencing data presented in this study are deposited in the EVA repository, accession number 'PRJEB82720'. The protein structural models generated in this study are provided in the **Supplementary Material**. KEL alleles are publicly available at https://www.isbtweb.org/resource/006kel.html and https://gnomad.broadinstitute.org/.
