# Supplementary material for: A structure-based in silico analysis of the Kell blood group system
Source: Front Immunol. 2024 Dec 6;15:1452637. doi: 10.3389/fimmu.2024.1452637 (PMC11669894; doi:10.3389/fimmu.2024.1452637)
Supplement: Supplementary file 1 [file DataSheet1.zip › Data Sheet 1/Supplementary Material Sections 1-12.PDF]

Supplementary Material

A Structure-Based *in silico* Analysis of the Kell Blood Group System

Gabriele Mayr[1†], Maike Bublitz[2†*], Tim Steiert[1], Britt-Sabina Loescher[1], Michael Wittig[1], Hesham El Abd[1], Christoph Gassner[1,2††], Andre Franke[1††*]

[1]Institute of Clinical Molecular Biology, University Hospital Schleswig-Holstein (UKSH) & Christian-Albrechts-University of Kiel, Kiel, Germany
[2]Institute of Translational Medicine, Faculty of Medical Sciences, Private University in the Principality of Liechtenstein (UFL), Triesen, Principality of Liechtenstein
[†] These authors contributed equally to this work and share first authorship
[††]These authors share senior authorship

*** Correspondence:**
Corresponding Authors:
Andre Franke: a.franke@ikmb.uni-kiel.de; Maike Bublitz: maike.bublitz-meier@ufl.li

Supplemental Results

1 Validation of the Kell Structural Model

Several key analyses carried out in this study relied on a 3D structural model of the Kell protein generated with Alphafold3. In order to validate the model, we generated an independent, template-based model with MODELLER and then compared the two models with respect to geometry, similarity and homology to available experimentally determined homologous structures.

Comparison of Kell Structural models generated with AlphaFold3 and MODELLER (shared residues 79-723)
Model geometry (Molprobity.org)

	AlphaFold3	MODELLER
Ramachandran outliers	0	10
Poor rotamers	2	15
Bad bonds	0	2
Bad angles	10	105
Cis non-prolines	0	1
C-alpha geometry outliers	2	15

TM-Score AlphaFold3 vs. MODELLER

```
*************************************************************************
***
 *                                 TM-SCORE
*
 * A scoring function to assess the similarity of protein structures
*
 * Based on statistics:
*
 *       0.0 < TM-score < 0.17, random structural similarity
*
 *       0.5 < TM-score < 1.00, in about the same fold
*
 * Reference: Yang Zhang and Jeffrey Skolnick, Proteins 2004 57: 702-710
*
 * For comments, please email to: zhng@umich.edu
*

*************************************************************************
***
```

Structure1: Kell_79-732_MODELLER   Length= 654
Structure2: Kell_79-732_AlphaFold3   Length= 654 (by which all scores are normalized)
Number of residues in common= 654
RMSD of the common residues= 4.533

TM-score   = 0.8559 (d0= 8.88)
MaxSub-score= 0.4859 (d0= 3.50)
GDT-TS-score= 0.5684 % (d<1)=0.2034 %(d<2)=0.3976 %(d<4)=0.7263 %(d<8)=0.9465
GDT-HA-score= 0.3559 % (d<0.5)=0.0963 %(d<1)=0.2034 %(d<2)=0.3976 %(d<4)=0.7263

Superposition in the TM-score: Length(d<5.0)=539 RMSD= 2.75

Structural similarity between Kell structural models and available homologous structures:
Kell models generated with AlphaFold3 or MODELLER were overall very similar (all-C-alpha RMSD <3.0 A^2, TM-score 0.86, GDT-TS Score 0.57). Structural differences observed were mainly caused by a rigid-body shift between MPD and MDD domains. The individual MPD and MDD domains of the two models differed by RMSDs of less than 1.7 A^2.
Both models were also in good agreement with experimentally determined homologous structures, particularly on the domain level (RMSDs between 0.26 and 2.95 A^2)
Between Kell and the available structurally determined homologues, *sequence identity* was moderate (22.2 – 31.2 %), but *sequence similarity* was high (38.8 – 45.6 %). The same was true between the three homologs, both on the sequence level (31 – 39.3 % identity; 43 – 55.8 % similarity) and also between the experimental structures (all-C-alpha RMSDs of 1.27 – 1.88 A^2).
RMSD values (A^2) were determined with PyMOL, as follows:
all C-alpha: Kell residues 79-732
MPD: Kell residues 79-137, 420-491 and 529-732
MDD: Kell residues 138-419 and 492-528

All C-alpha RMSD	Kell Alphafold3 79-732	Kell MODELLER 79-732	3dwb	1dmt	3zuk chain A
Kell Alphafold3 79-732	0	2.99	2.68	3.73	3.66
Kell MODELLER 79-732		0	0.42	1.04	1.46
3dwb (human ECE-1)			0	1.35	1.46
1dmt (human neutral endopeptidase)				0	1.35
3zuk chain A (*M. tuberculosis* ZMP1)					0

All C-alpha RMSD	Kell Alphafold3 MPD	Kell MODELLER MPD	3dwb MPD	1dmt MPD	3zuk chain A MPD
Kell Alphafold3 MPD	0	1.26	1.26	1.41	1.45
Kell MODELLER MPD		0	0.33	0.65	0.71
3dwb MPD (human ECE-1)			0	0.91	0.76
1dmt MPD (human neutral endopeptidase)				0	0.89
3zuk chain A MPD (*M. tuberculosis* ZMP1)					0

All C-alpha RMSD	Kell Alphafold3 MDD	Kell MODELLER MDD	3dwb MDD	1dmt MDD	3zuk chain A MDD
Kell Alphafold3 MDD	0	1.66	1.39	2.77	2.95
Kell MODELLER MDD		0	0.26	1.44	1.84
3dwb MDD (human ECE-1)			0	1.28	1.27
1dmt MDD (human neutral endopeptidase)				0	1.88
3zuk chain A MDD (*M. tuberculosis* ZMP1)					0

Sequence similarity of KELL homologues with available structures:

Sequence ID (%)	Kell	human ECE-1	human neutral endopeptidase	*M. tuberculosis* ZMP1
Kell	100	31.22	24.29	22.19
human ECE-1		100	39.30	31.03
human neutral endopeptidase			100	31.00
M. tuberculosis ZMP1				100

Sequence Similarity (%; Bioinfornmatics.org)	Kell	human ECE-1	human neutral endopeptidase	*M. tuberculosis* ZMP1
Kell	100	45.59	38.8	44.81
human ECE-1		100	55.87	43.00
human neutral endopeptidase			100	44.55
M. tuberculosis ZMP1				0

Similarity grouping: G, AVLI, FYW, CM, ST, KRH, DENQ, P

2 Structural conservation of Kell variants among the four classes

During the course of evolution, amino acids in proteins are continuously substituted if dispensable for function or stability, and therefore, conserved amino acid positions are generally considered more important than variant sites. Conserved positions that are still observable among very remote homologs and are often essential for the stability of a shared protein fold. Such structurally conserved residues are usually buried inside the hydrophobic core of the proteins and often in close contact with other invariant sites, and a mutation in such positions can have a globally destabilizing effect, preventing proper protein folding. Mutation may also have a local destabilizing effect only, diminishing the levels and/or the lifetime of functional protein rather than abrogating its production entirely.

Fig. S2 shows the distribution of structural conservation of the KEL variant classes with up to three paralogs. The NullV dataset includes the most structurally conserved amino acids with only one variant site that is not conserved in any of the three paralogs, while the other variant classes show a similar distribution with several non-structurally conserved sites.

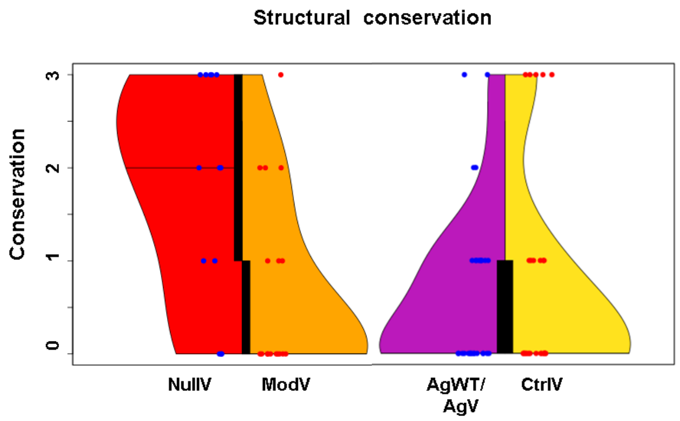

Supplemental Figure S2. Distribution of structural conservation of Kell variant residues among the three paralogs with known protein structures in the M13 protein family: Human ECE-1 (PDB ID 3dwb) and NEP (PDB ID 1dmt), and Zmp1 from *Mycobacterium tuberculosis* (PDB ID 3zuk)

3 Distribution of physico-chemical property scores

In the wildtype Kell protein, residues from the different variant classes show significant differences from each other, reflecting the pre-dominant locations of the classes NullV, ModV, AgWT/AgV and CtrlV (**Supplemental Fig. S3-1** showing results for Polarity only, left side of split violin plots. FigS3 is complementary to Fig. 3 in the main manuscript that shows the distribution for hydrophobicity and side chain volume scores). However, these properties are much more uniformly distributed when comparing to the variant amino acids after mutation (right side of split violin plots). This demonstrates that the physico-chemical properties of variant amino acids fit less well into their respective structural environment than the wildtype amino acids.

Most visible is the difference for hydrophobicity and polarity: While wildtype values (**Supplemental Fig. S3-2,** left panel) are very characteristic for the respective dataset categories, variant values are distributed almost equally among the subsets (**Supplemental Fig. S3-3,** right panel). The stronger hydrophobicity in the NullV and ModV wildtype amino acids reflect the buried and partly buried location of NullV and ModV. Less hydrophobicity of AgWT/AgV is typical for their mostly exposed positions. CtrlV are found in all locations, buried and exposed, and this is reflected in the distribution as well. Similarly, the distribution of wildtype polarity is very specific for each subgroup and more uniform in the variants. Changes in side chain volume are most visible in NullV and ModV. Substitutions to smaller side chains result in loss of buried intramolecular interactions, and if increasing, lead to distortions due to the lack of space in the densely packed core. Both 'directions' of volume change therefore globally destabilize the fold.

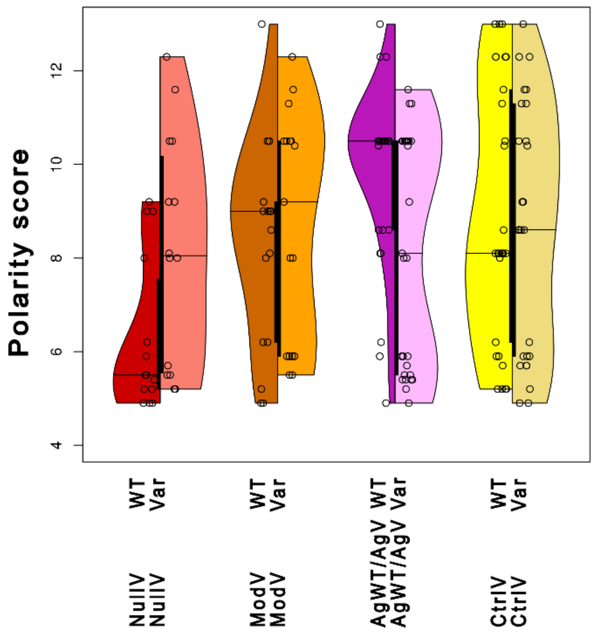

Supplemental Figure S3-1: Distribution of values for polarity of amino acids in variant sites of the Kell protein. Variants of the categories NullV, ModV, AgWT/AgV and CtrlV (right and lighter side of each violin plot) were each compared to their corresponding wild type amino acids (left and darker side). Differences before and after mutation are larger for NullV, ModV and AgV compared to CtrlV, that shows very similar distributions.

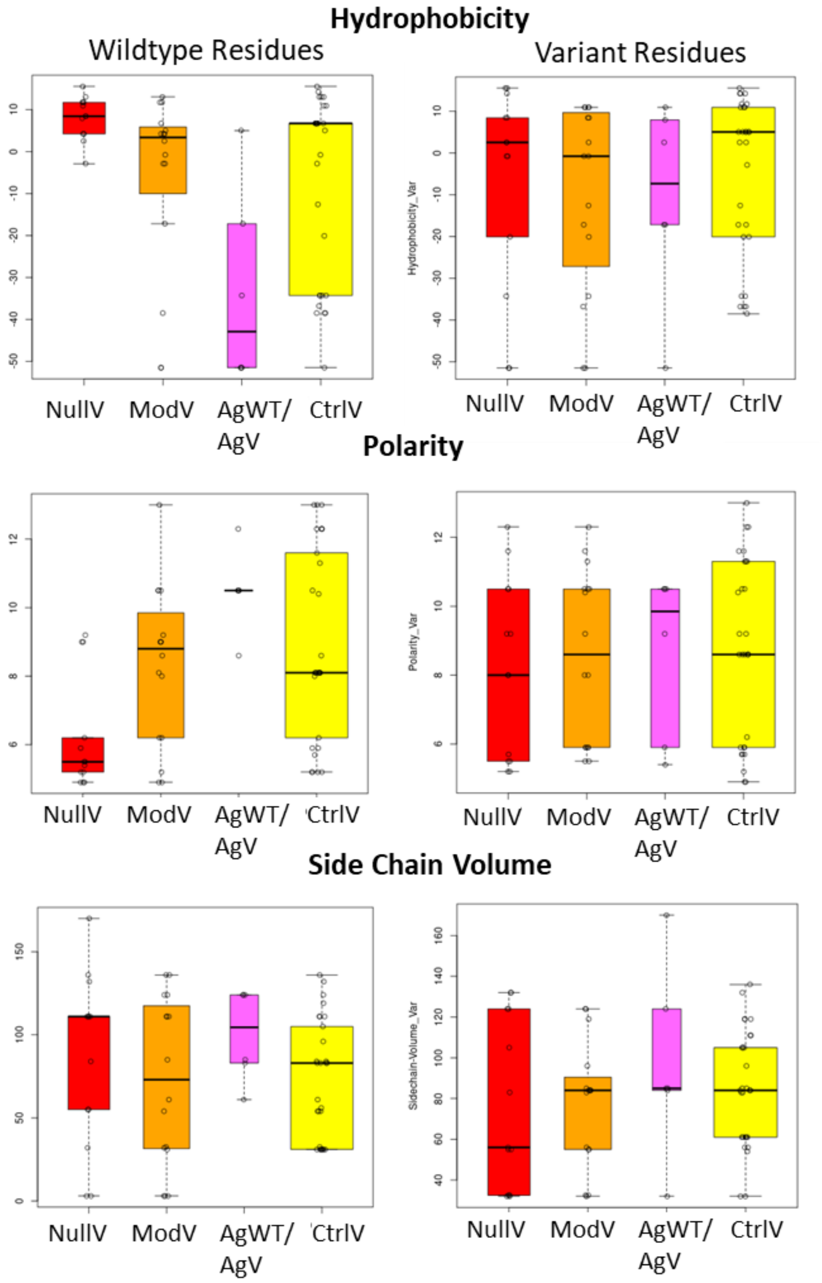

Supplemental Figure S3-2: Hydrophilicity, polarity and side chain volumes of the different variant classes of the Kell protein. The distribution of values for the wildtype residues of the classes NullV, ModV, AgWT/AgV and CtrlV (left) differ significantly from each other, whereas variant residues (right) resulting from mutation have a more uniform distribution of property values.

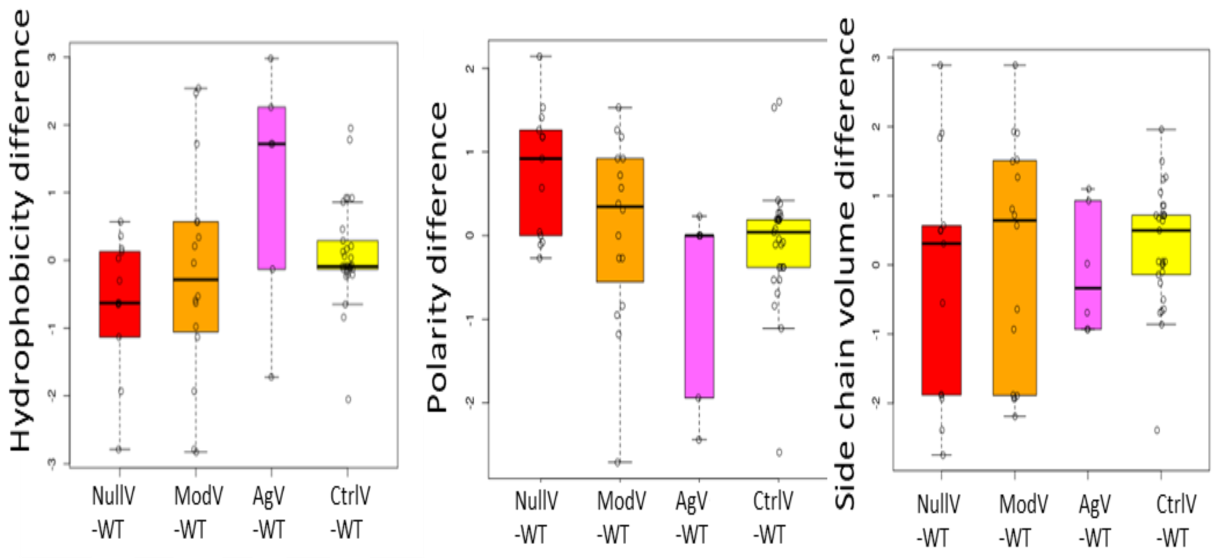

Supplemental Figure S3-3: Differences in Hydrophobicity, polarity and side chain volumes of all Kell protein variant residues, compared to wildtype. Values for the categories NullV, ModV, AgWT/AgV and CtrlV were each subtracted from their corresponding wild type amino acid values and normalised.

4 Analysis of dataset Kell variations by the dbNSFP metaserver

The dbNSFP database provides precalculated functional prediction and annotations of all potential non-synonymous single-nucleotide variants in the human genome and includes data from more than 40 different sources that predict the potential deleterious effect of genetic variants as well as diverse conservation scores and other functional annotations. The Webservice is routinely applied to filter and prioritize mutations. We therefore collected all available scores for the Kell variation dataset (**Supplemental Table S3**) and visualized the distribution of scores in violin plots (**Supplemental File dbNSFP_Violinplots.zip**). Results data for all Kell protein sequence variants provided by the dbNSFP database are stored in **Supplemental Table S7.**

Most pathogenicity prediction methods provided by the dbNSFP metaserver distinguish well between NullV, ModV, AgWT/AgV and CtrlV, showing the same trends as in described in the course of this work. We would like to highlight the results of MPC [preprint reference only: https://doi.org/10.1101/148353] that among pathogenicity prediction methods distinguished ideally between the dataset classes. (**Supplemental Fig. S5A**), with all NullV mutations predicted to be pathogenic as well as almost all of the ModV mutations, while scores for AgWT/AgV and CtrlV are lower and among the two almost similarly distributed. Among CtrlV, the highest MPC score show Phe431Ser, Ala645Thr and Ala645Glu.

5 Protein structural destabilization prediction of Kell variants

We collected predictions of structural destabilization for every mutation by applying MaestroWeb and RaSP to the structural model of KEL as described in the main manuscript (**Supplemental Fig. S5 B,C**). Overall, both methods applied are agreeing in the result: The strongest predicted destabilizing effect have NullV, less affecting the structure are ModV, while the AgWT/ have the least consequence, as well as CtrlV. The most destabilizing and pathogenicity scores in the CtrlV are reached Phe431Ser and Ala645Glu/Thr (prediction scores in **Supplemental Table S3**). Interestingly, Ala645Val, another variant at the latter position, is a KEL1 ModV together with Thr193Met (*KEL**01M.05). A visual inspection of the positions the structural model confirmed, that these positions are likely destabilizing by introducing hydrophilic amino acids into a tightly packed and conserved hydrophobic environment.

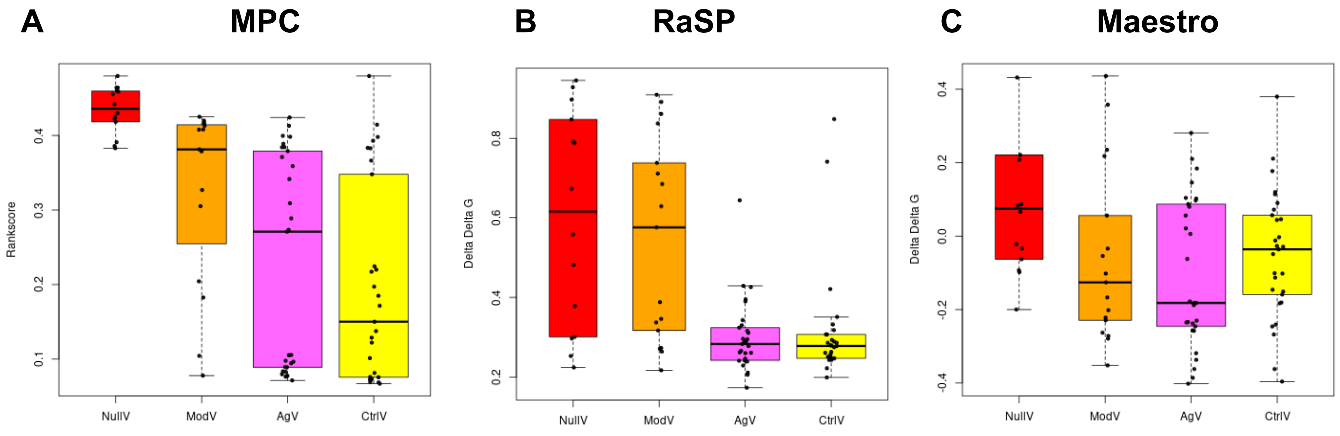

Supplemental Figure S5: Distribution of pathogenicity scores by **(A)** MPC derived from the dbNSFP webserver, which is the method that reflects best the predicted destabilizing effect of KEL variants, as also is viewed in **(B)** Prediction of destabilization of KEL variants by RaSP and **(C)** Maestro.

6 Epitope predictions by Discotope

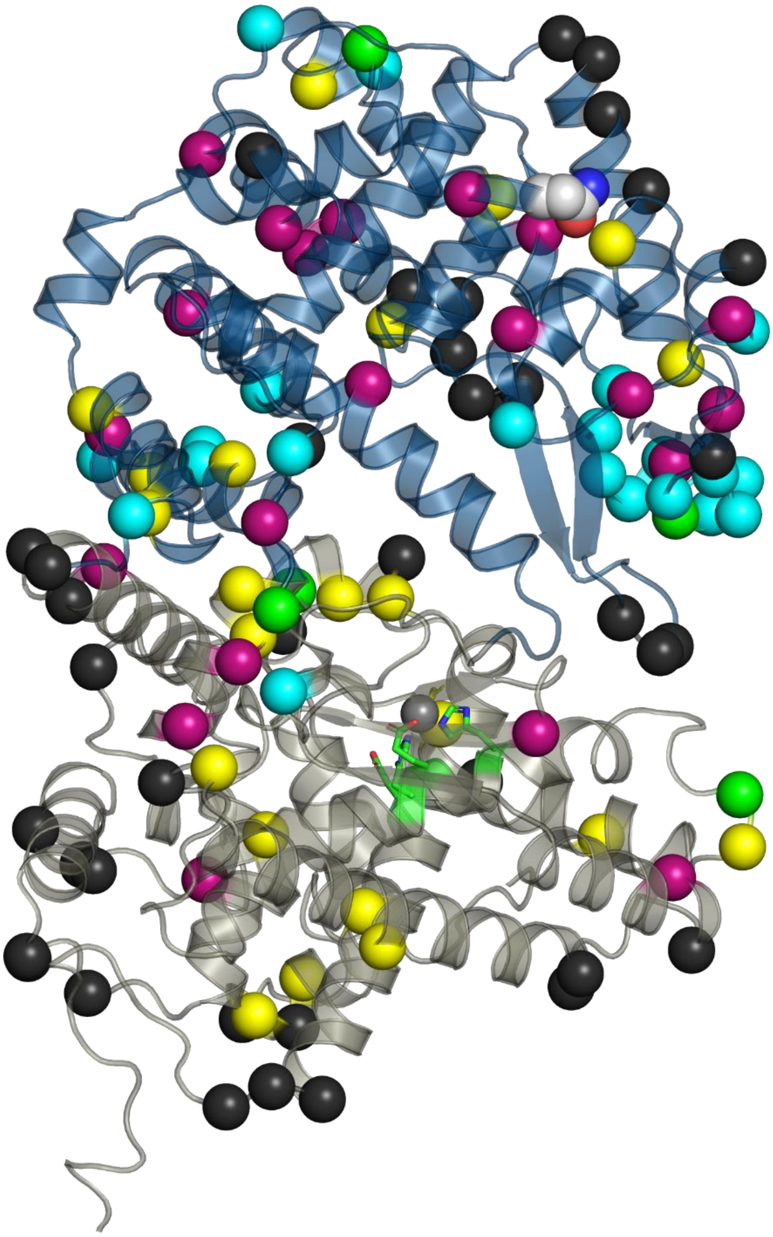

Supplemental Figure S6: 3D localization of conformational epitope predictions by Discotope showing random distribution of predicted antigenic sites (confidence threshold set at 'high'). In spheres representation the CA atoms of selected amino acids. AgWT/AgV and CtrlV are highlighted in magenta and yellow. In green, five AgWT/AgV positions were predicted by Discotope. In Cyan, Discotope epitope predictions that agree with manual epitope predictions as described previously (main manuscript **Fig. 5**). In dark grey, other predicted Discotope epitope predictions, of which many locate membrane-proximal and are therefore very unlikely antigenic. Interestingly, Discotope did not predict epitopes at the most top region of the membrane-distal domain. Active site in green sticks and Zn^{2+} in grey. The *N*-glycosylation site in grey spheres, with nitrogen in blue and oxygen in red.

7 Epitope predictions by BepiPred

Confidence threshold level: Moderate (top 50%)

MEGGDQSEEEPRERSQAGGMGTLWSQESTPEERLPVEGSRPWAVaRRvltaililglllcfsvllfYNFQ
NCGPRPCETSvcLDlRDHylasgnTSvapctdffsfacgRaKETNNsfQElaTknKNRlRRilEVQNSWHPG
SgeEkafQfynscmdtLAiEAAgtGplRQviEElggwRisGKWTSLNfnRtlRLlmsQyghFpffRaYlgPHPAS
PHTPviqidqPEfDVPLKQDQEQKIYAQIFREyLTylNQlgTllggDPSKvQEhsSLsiSitSRlFQFlRPLE
QRRAQGKLFQMvTiDQlKEMaPAidwLsclQatfTPMSlSPSqSlvvHdvEylKNmsQlvEEMlLKQRdfl
qshmilglvvtlsPaldSQfQEarRKlSQKLRELTEQPPMPARPRWMKcvEEtgTffeptlaalfvREafgPStRSa
aMKlfTairDaliTrlRnlPWmnEEtqNMaqDkvAQlQvEmgasEWalKPELaRQeyNDiQlgSSflQsvlscvRs
lRARIvQSfLQPHPQHRWKVSpwDvnaYysVSDhvvvfpagllqPpffhPgypravnfgaagsimahellhifyQllL
PGGcLaCDNHalQEahLclkRhyaAfPlPSRTSfNdsltflenaadvgglaialqaySKRLLRHHGETVlPSlDlsp
qqiffrsyaqvmcRKPSPQDSHDTHsppHlrvhgplsstPafaRyfRcaRGaLlnpSSRcQlw

8 Occurrence of specific amino acids in antigenic and non-antigenic positions

The tolerance of some conserved CtrlV mutations is surprising from a structural point of view, especially if they are buried and in close vicinity of NullV or ModV, as are Thr421Met, Phe431Ser, Ala574Thr and Ala645Glu/Thr. Possibly, some specific amino acids are generally less immunogenic, for example, threonine, methionine, lysine and asparagine are not typically observed in antigenic positions (AgWT and AgV), but each of them is more than 5-fold more common in in the CtrlV dataset than in the overall Kell sequence. On the other hand, arginine, glutamine and tryptophan residues are overrepresented in the AgWT/AgV class (**Supplemental Fig. S8**).

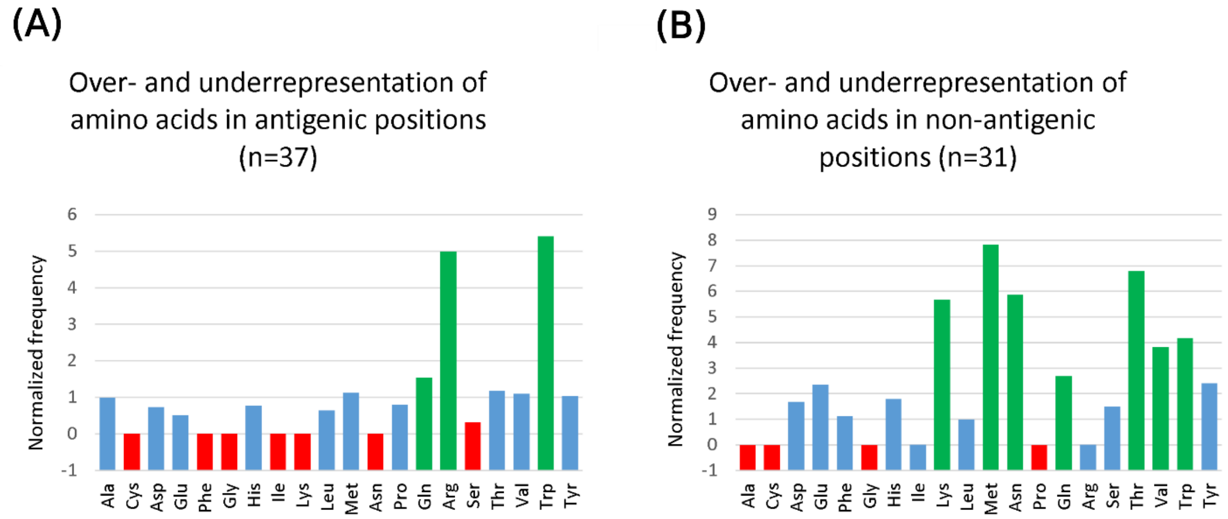

Supplemental Figure S8: Over- and underrepresentation of individual amino acids in antigenic and non-antigenic positions. (A) Histogram of amino acid occurrence in antigenic positions (n=37). Values were normalized against each amino acid's occurrence in the total Kell protein sequence. **(B)** Histogram of amino acid occurrence in non-antigenic positions (n=31). Values were normalized against each amino acid's occurrence in the total Kell protein sequence.

9 HLA binding predictions of variant Kell peptides

For a robust antibody production, B cells need to receive a co-stimulatory signal from $CD4^+$ T cells. This signal is usually in the form of cytokines that antigen-specific T cells secrete after it recognizes its cognate peptide-HLA-II complexes on the surface of B cells. HLA-II proteins present variable-length linear peptides that are mainly between 13 to 17 amino acids long with 15 being the mode. The genes encoding HLA-II proteins, namely, *HLA-* [*DRA*, *DRB*, *DQA*, *DQB*, *DPA*, *DPB*] are highly polymorphic with *HLA-DRA* which encodes the alpha-chain of the HLA-DR protein being the only exception that is monomorphic. Nonetheless, *HLA-DRB* genes, particularly, *DRB1*, which encode the beta chain of the HLA-DR protein exhibit an astronomical degree of variability with thousands of alleles already reported. To investigate the impact of HLA-genetic variability on the presentation of peptides encoded by different KEL alleles, 15-mer peptides with Kell antigenic variants (AgV, AgWT) as well as non-antigenic Kell variants (CtrlV) at all possible positions 1-15 were submitted to the peptide-immune annotation (PIA) to computationally estimate their presentation probability, *i.e.* their likelihood of presentation by the corresponding *HLA-DRB1* alleles. Different trends were observed across the 19 *HLA-DRB1* alleles tested, however, no significant difference between the different groups was observed among the test alleles (**Supplemental Fig. S9**).

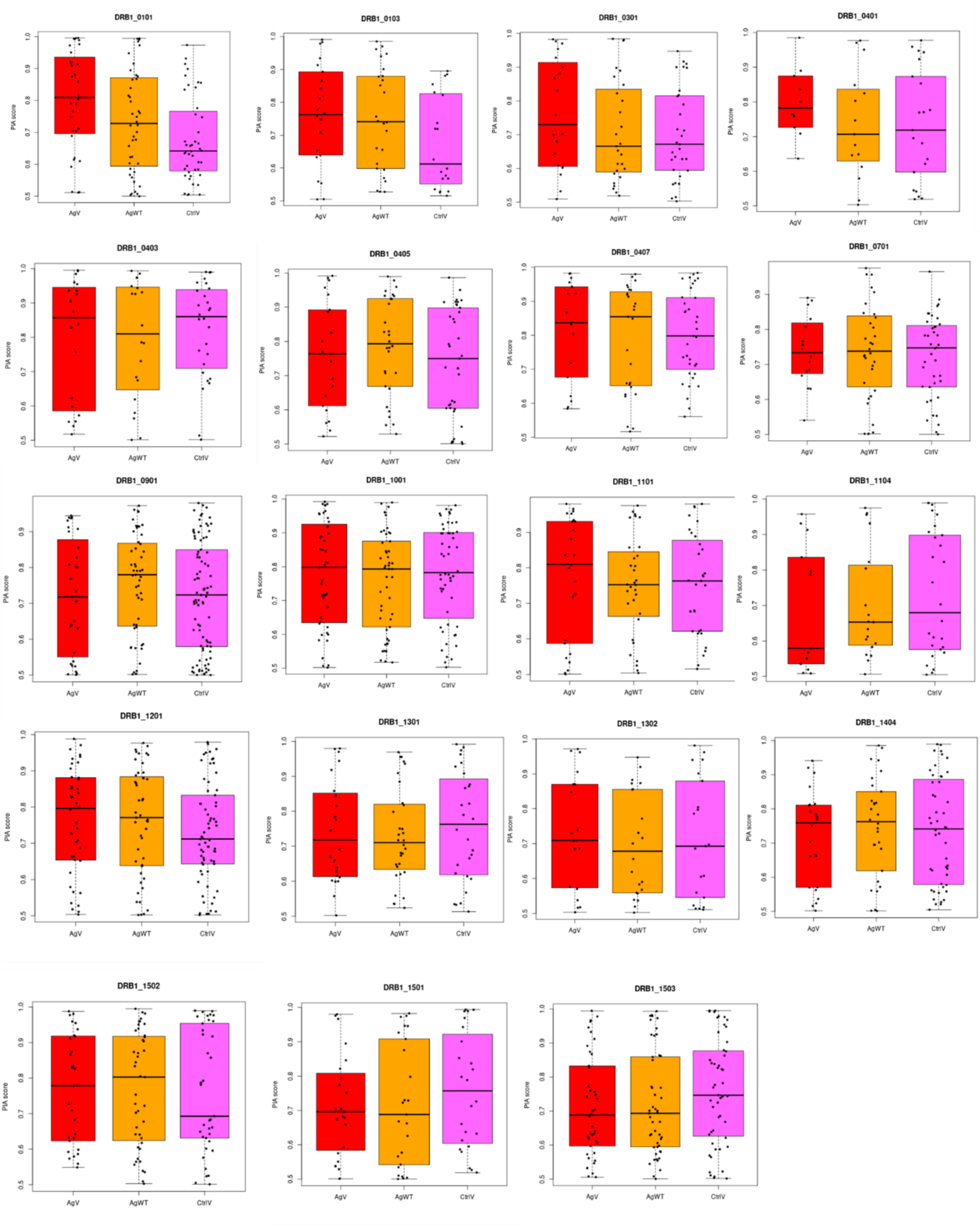

Supplemental Figure S9: HLA binding predictions using PIA. Only scores above 0.5 were plotted. A score above 0.9 can be considered as significant.

10 Prediction of potential new destabilizing and antigenic variants from whole exome DNA sequencing data

We searched for all Kell missense variants in 20,563 exomes from an in-house whole exome sequencing dataset (**Supplemental file Table S5**) and filtered potential Kell destabilizing (K_0) and antigenic (AgV) variants using a criteria catalog (see main manuscript). An example for predicting a Kell phenotype is depicted in **Supplemental Fig. S10-1**. With this semi-automated approach, we detected 19 potentially critical Kell variants (**Supplemental Table S6**). They are localizing in 3D similar to AgWT/AgV mostly in the membrane-distal domain (**Supplemental Fig. S10-2**).

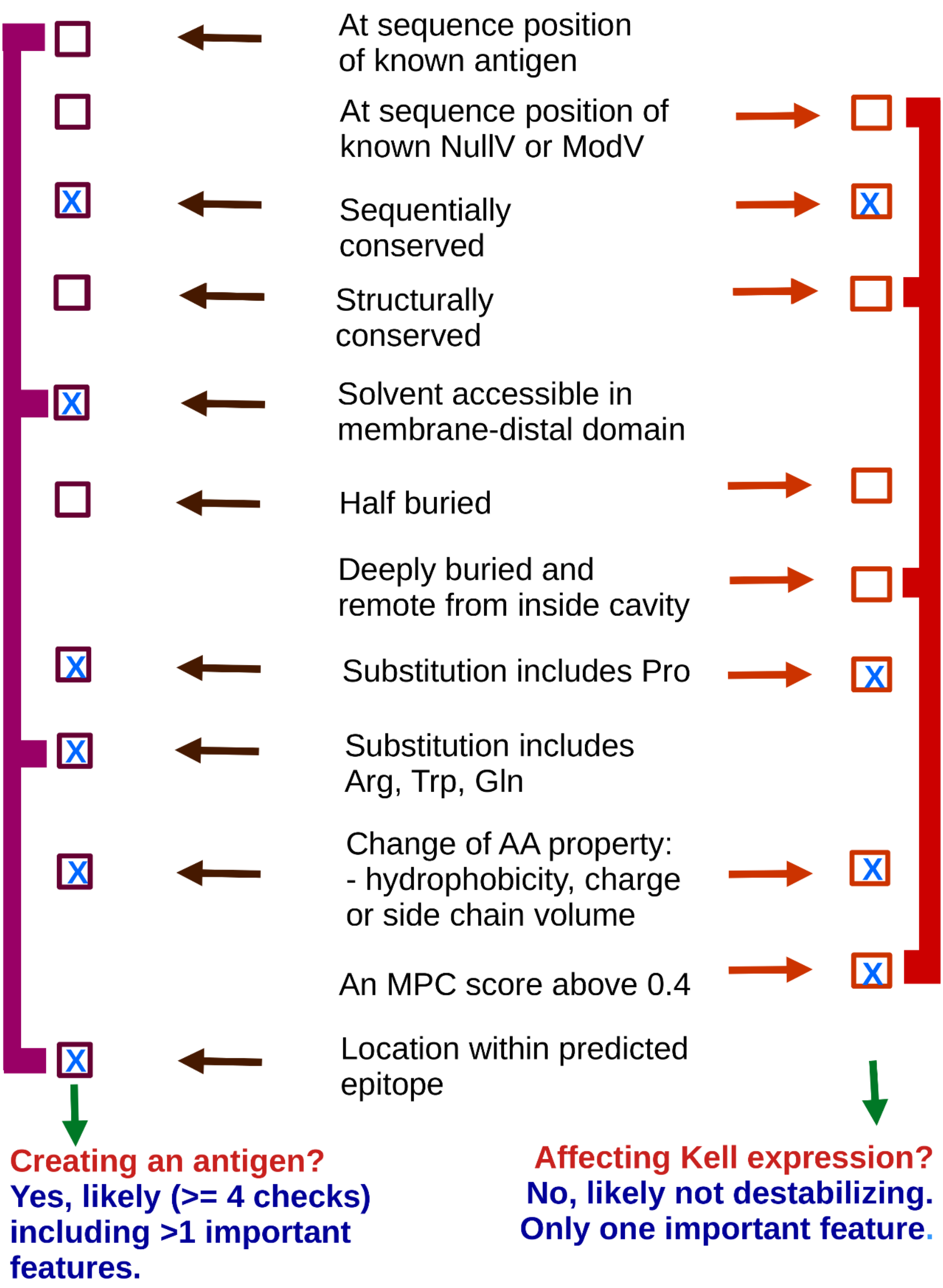

Supplemental Figure S10-1: New Kell variant phenotype prediction on the whole exome sequencing variant Gln245Pro (**Supplemental Table S6**) based on conditional filters derived from parameters observed to be relevant for antigenicity and Kell expression. Left column of boxes indicate properties of potential AgWT/AgV, right panel, indicate properties of destabilizing variants. Boxes can be checked if the condition in the same line fits the variant under observation. Missing boxes on either side of the panel point to conditions that are not relevant for the group. Brackets indicate the most relevant combinations.

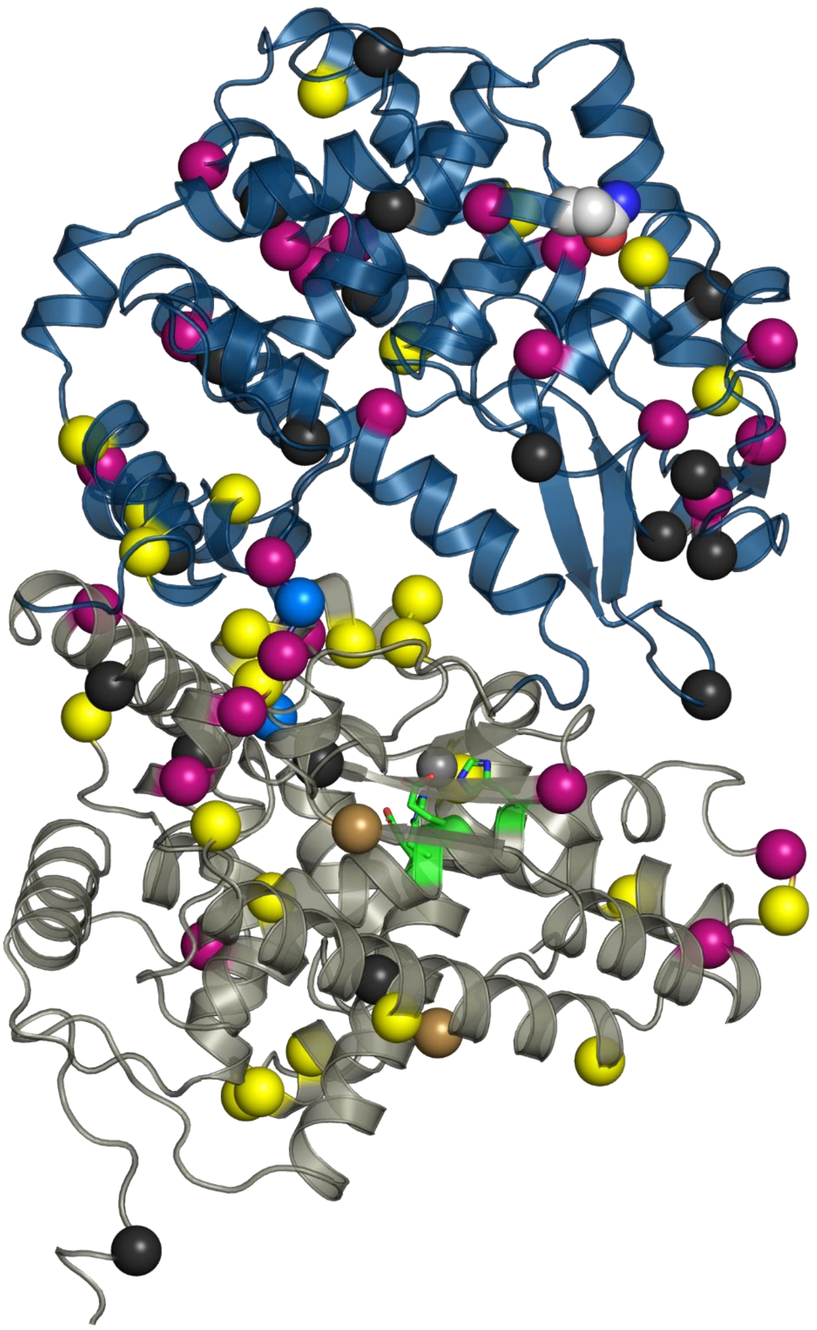

Supplemental Figure S10-2: 3D localization of potentially destabilizing or immunogenic Kell variants from an in-house exome sequencing project. The distribution of selected amino acid positions (black, from the exome sequencing, in brown from Howe&Stack, Transfusion 2023, see also **Supplementary Table S6**) resembles the preferred localization of AgWT/AgV (magenta) in the membrane-distal domain (blue), while CtrlV (yellow) are more randomly distributed over both subdomains, the membrane-proximal (grey) and the membrane-distal domain. The N-glycosylation site is indicated as grey spheres, nitrogen in blue and oxygen in red. The active site is shown as green sticks and the Zn^{2+} as grey sphere.

11 Kell protein AgWT/AgV and predicted epitope sequence positions

```
1
MEGGDQSEEE PRERSQAGGM GTLWSQESTP EERLPVEGSR PWAVARRVLT
51
AILILGLLLC FSVLLFYNFQ NCGPRPCETS VCLDLRDHYL ASGNTSVAPC
101
TDFFSFACGR AKETNNSFQE LATKNKNRLR RILEVQNSWH PGSGEEKAFQ
151
FYNSCMDTLA IEAAGTGPLR QVIEELGGWR ISGKWTSLNF NRTLRLLMSQ
201
YGHFPFFRAY LGPHPASPHT PVIQIDQPEF DVPLKQDQEQ KIYAQIFREY
251
LTYLNQLGTL LGGDPSKVQE HSSLSISITS RLFQFLRPLE QRRAQGKLFQ
301
MVTIDQLKEM APAIDWLSCL QATFTPMSLS PSQSLVVHDV EYLKNMSQLV
351
EEMLLKQRDF LQSHMILGLV VTLSPALDSQ FQEARRKLSQ KLRELTEQPP
401
MPARPRWMKC VEETGTFFEP TLAALFVREA FGPSTRSAAM KLFTAIRDAL
451
ITRLRNLPWM NEETQNMAQD KVAQLQVEMG ASEWALKPEL ARQEYNDIQL
501
GSSFLQSVLS CVRSLRARIV QSFLQPHPQH RWKVSPWDVN AYYSVSDHVV
551
VFPAGLLQPP FFHPGYPRAV NFGAAGSIMA HELLHIFYQL LLPGGCLACD
601
NHALQEAHLC LKRHYAAFPL PSRTSFNDSL TFLENAADVG GLAIALQAYS
651
KRLLRHHGET VLPSLDLSPQ QIFFRSYAQV MCRKPSPQDS HDTHSPPHLR
701
VHGPLSSTPA FARYFRCARG ALLNPSSRCQ LW
```

Supplemental Figure S11: Sequential annotations of the Kell protein. Magenta: AgWT/AgV wildtype positions. Other similar colors indicate predicted conformational epitopes, that are composed of regions adjacent in 3D. Green: Intracellular domain 1-43. Brown: Transmembrane domain 44-67. Underlined: The membrane-distal domain (MDD), not underlined: membrane-proximal domain (MPD). Cysteines forming a disulfide bridge in yellow, active site in blue characters, N-glycosylation site N191 in grey.

12 SUPPLEMENTAL METHODS

Analysis of amino acid variants on the 3D structural level

Some general concepts about protein structural biology are relevant for the analysis of protein variations: Every amino acid is unique in its properties, and these determine the structural and functional role of the residue within a protein structure. Protein folding is driven by thermodynamics, reaching a global free energy minimum in a stable 3D conformation, and causes the protein chain to fold, generating a network of covalent and non-covalent interactions. As a result, hydrophobic amino acids prefer to locate inside the molecule ('buried') and shielded from the solvent, ideally with van-der-Waals contacts to other hydrophobic amino acids. Hydrophilic and polar amino acids are more often exposed on the surface of the protein. If buried, they form hydrogen bonds or stronger ionic bonds (salt bridges) if charged, to stabilize the structural core. Conserved salt bridges and di-sulfide bonds are often responsible for global protein stability by connecting secondary structure elements and stabilizing loop conformations. Besides hydrophobicity which determines the preference of an amino acid to be on the surface or in a buried location, other properties may be important, such as side chain volume and flexibility: Some amino acids are small and allow sharp turns in the protein backbone, while others are large and bulky and therefore much more constrained in their flexibility. A special case is proline since its side chain is covalently linked to the backbone and therefore very conformationally very limited. In alpha helices a substitution to proline is assumed to disrupt the structural stability, while loops may adapt easier to this change. Mutations to larger or smaller side chain volumes may cause steric clashes in a densely packed environment and following destabilize the protein locally or globally. It is therefore commonly assumed that amino acid substitutions are less likely to have an influence on expression and function of the protein if the properties of wildtype and variant amino acid side chains are similar. In this work, the similarity between amino acids was assessed using the following physico-chemical property values as derived from the AAIndex database:

Hydrophobicity (AAIndex database accession PRAM900101)

A/L	R/K	N/M	D/F	C/P	Q/S	E/T	G/W	H/Y	I/V
-6.7	51.5	20.1	38.5	-8.4	17.2	34.3	-4.2	12.6	-13
-11.7	36.8	-14.2	-15.5	0.8	-2.5	-5	-7.9	2.9	-10.9

Polarity (AAIndex database accession GRAR740102)

A/L	R/K	N/M	D/F	C/P	Q/S	E/T	G/W	H/Y	I/V
8.1	10.5	11.6	13	5.5	10.5	12.3	9	10.4	5.2
4.9	11.3	5.7	5.2	8	9.2	8.6	5.4	6.2	5.9

Sidechain-Volume (AAIndex database accession GRAR740103)

A/L	R/K	N/M	D/F	C/P	Q/S	E/T	G/W	H/Y	I/V
31	124	56	54	55	85	83	3	96	111
111	119	105	132	32.5	32	61	170	136	84
