# Supplementary material for: A structure-based in silico analysis of the Kell blood group system
Source: Front Immunol. 2024 Dec 6;15:1452637. doi: 10.3389/fimmu.2024.1452637 (PMC11669894; doi:10.3389/fimmu.2024.1452637)
Supplement: Supplementary file 1 [file DataSheet1.zip › Data Sheet 1/Supplement_dbNSFP.ZIP!/Supplement_dbNSFP/REVEL.pdf]

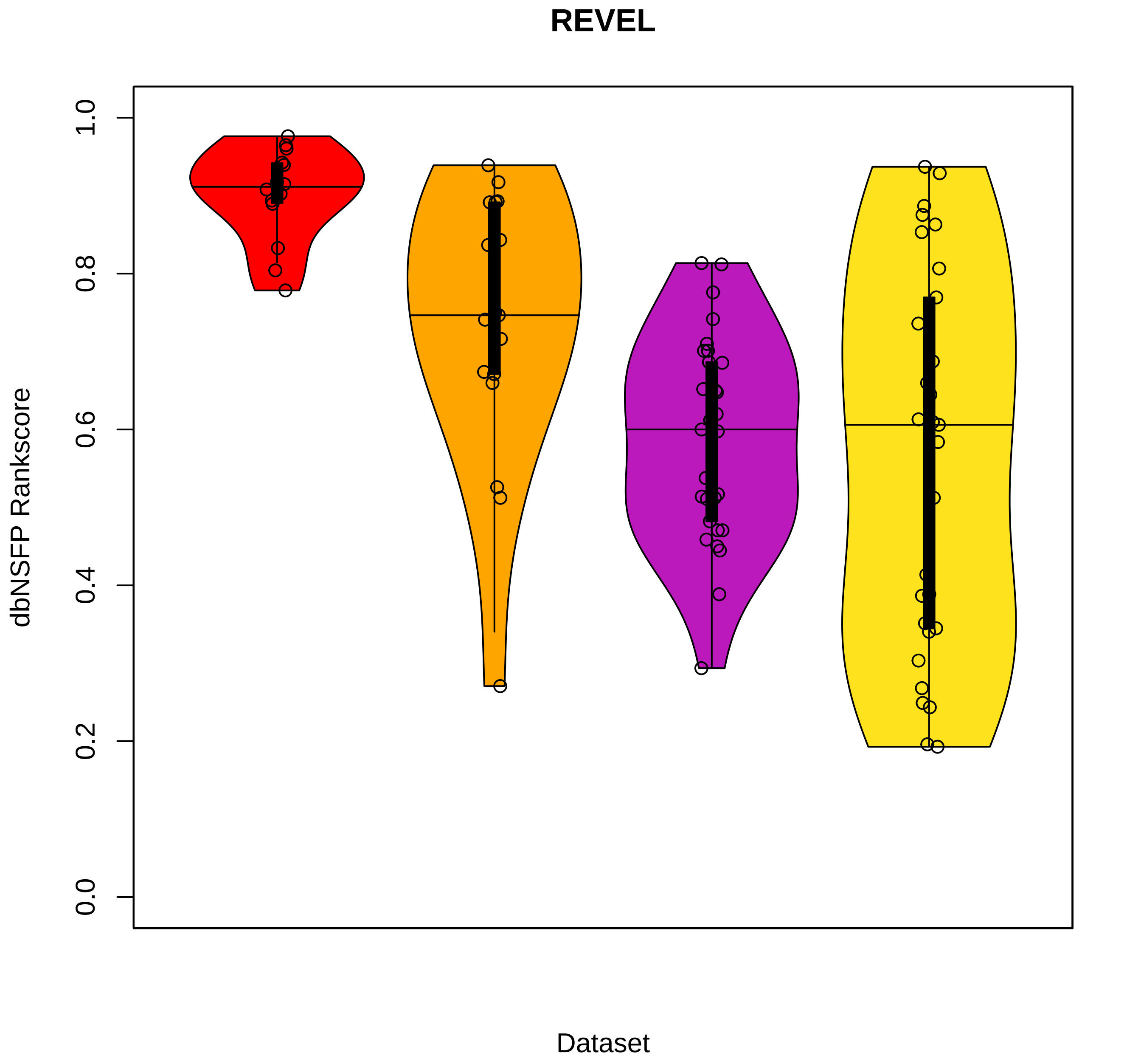

REVEL
dbNSFP Rankscore
0.0
0.2
0.4
0.6
0.8
1.0
Dataset
